# Anionic Liposomes as Optimal Membrane Fusion Carriers Enabling in Situ Multiplexed Detection of Extracellular Vesicle MicroRNAs

**DOI:** 10.1002/advs.202519758

**Published:** 2025-12-08

**Authors:** Jing‐Yuan Ma, Xiao Wang, Yuhan Cai, Guancheng Wang, Mingze Lu, Kaizheng Feng, Ying Zhao, Xue Wu, Xiaoping Zhang, Haoan Wu, Wei Yu, Ming Ma, Zheng Ge, Yu Zhang

**Affiliations:** ^1^ State Key Laboratory of Digital Medical Engineering Jiangsu Key Laboratory for Biomaterials and Devices School of Biological Science and Medical Engineering Southeast University Nanjing Jiangsu 210096 P. R. China; ^2^ Department of Hematology Zhongda Hospital School of Medicine Institute of Hematology Zhongda Hospital Southeast University Nanjing Jiangsu 210009 P. R. China; ^3^ Department of Medical Laboratory Taikang Xianlin Drum Tower Hospital Nanjing University School of Medicine Nanjing Jiangsu 210000 P. R. China

**Keywords:** extracellular vesicles, liposomes, liquid biopsy, membrane fusion, microRNA detection

## Abstract

Extracellular vesicle (EV) microRNAs (miRNAs) are promising liquid biopsy biomarkers for non‐invasive diagnosis, monitoring, and therapeutic evaluation of cancer. However, sensitive EV miRNA detection is hindered by complex pre‐analytical processing. Here, the authors present an anionic liposome (AL) assisted membrane fusion strategy enabling one‐step multiplexed quantification of EV miRNAs directly from plasma without EV isolation or RNA extraction, termed EValarm (Anionic Liposome Assisted miRNAs Monitoring for Extracellular Vesicles). Liposomes encapsulating probes are prepared using a microfluidic chip, achieving catalytic signal amplification after target recognition of miRNA. Systematic lipid screening identified ALs as optimal carriers, exhibiting minimal background and superior sensitivity compared to cationic and neutral liposomes. The AL‐based assay delivered accuracy comparable to quantitative PCR with a streamlined workflow. Applied to 106 clinical samples from lymphoma patients and healthy controls, integration with artificial intelligence achieved high accuracy (AUC > 0.99). In summary, this study demonstrates a platform enabling direct and sensitive plasma EV miRNA detection, offering strong potential for clinical translation in cancer liquid biopsy.

## Graphical abstract

A novel strategy for in situ multiplexed miRNA analysis of plasma extracellular vesicles (EValarm) has been developed. EValarm enables straightforward detection of different extracellular vesicle miRNAs from plasma samples through systematic screening of liposome surface charges, without requiring complex pretreatment steps, and achieves satisfactory accuracy in lymphoma patient detection in combination with artificial intelligence analysis.

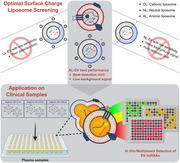

## 1 Introduction

Cancer remains one of the leading causes of death worldwide,^[^ ^1^, ^2^, ^3^ ^]^ and minimally invasive diagnosis, progression monitoring, and treatment evaluation are of significant importance in reducing cancer‐related mortality.^[^ ^4^ ^]^ Liquid biopsy has emerged as a promising diagnostic strategy due to its non‐invasive nature, simplified sample collection, and ease of processing, offering a rapid, low‐cost, and scalable approach for early cancer detection, thereby attracting broad attention.^[^ ^5^, ^6^ ^]^ Tumor‐derived extracellular vesicles (EVs) have gained increasing attention as potential biomarkers in liquid biopsy.^[^ ^7^, ^8^ ^]^ The molecular cargo encapsulated within EVs, such as microRNAs (miRNAs), is believed to reflect the physiological or pathological state of their cells of origin.^[^ ^9^, ^10^, ^11^ ^]^ The lipid bilayer membrane of EVs provides structural protection that shields miRNAs from degradation in biofluids.^[^ ^12^ ^]^ In addition, EVs derived from tumor cells often carry a distinct miRNA composition compared to those released by normal cells,^[^ ^13^, ^14^ ^]^ making EV miRNAs a promising class of biomarkers in liquid biopsy.^[^ ^15^, ^16^ ^]^ Of particular relevance, in malignant lymphomas, EV miRNAs, including miR‐155‐5p, miR‐125b‐5p, and miR‐21‐5p, have been widely recognized for their critical roles in monitoring disease onset and progression, evaluating therapeutic efficacy, and predicting patient prognosis.^[^ ^17^, ^18^, ^19^, ^20^, ^21^ ^]^

However, current EV miRNA detection methods have significant limitations, including complex workflows, time‐consuming processes, and high cost.^[^ ^22^, ^23^, ^24^ ^]^ In general, these methods require EVs to be first extracted from a complex body fluid environment. Then, the EVs have to be lysed to release the encapsulated miRNAs,^[^ ^25^ ^]^ and finally, signal amplification has to be performed using methods such as quantitative PCR (qPCR).^[^ ^9^ ^]^ Notably, both EV isolation and miRNA extraction steps resulted in sample loss.^[^ ^26^, ^27^, ^28^ ^]^ This is because miRNAs that have lost the protection of the phospholipid bilayer are easily degraded, leading to a decrease in the sensitivity and specificity of the measurement. In addition, time‐consuming and labor‐intensive separation and purification procedures hinder further applications.

To overcome the limitations of conventional EV miRNA detection, membrane fusion‐based approaches have been developed.^[^ ^29^ ^]^ These approaches employ naturally derived or synthetic lipid vesicles to encapsulate signal amplification systems,^[^ ^30^, ^31^, ^32^ ^]^ thereby enabling direct detection without compromising EV integrity. Membrane fusion, the process by which two separate vesicles with a lipid bilayer merge into a unified structure, plays a crucial role in many biological events, including EV secretion and intracellular communication.^[^ ^33^, ^34^ ^]^ This process enables not only the physical integration of membranes but also the mixing of respective contents,^[^ ^35^ ^]^ thereby facilitating delivery of detection systems into intact EVs while avoiding loss of miRNAs during extraction or interference from background nucleic acids.^[^ ^36^ ^]^

Among natural lipid vesicles, cell membrane vesicles (CMVs) have received much attention due to the ability to specifically recognize tumor‐derived EVs via homotypic fusion, enabling in situ detection of miRNAs.^[^ ^37^, ^38^ ^]^ Nonetheless, the preparation of CMVs is dependent on complex cell membrane separation and reconstruction processes, making large‐scale production difficult. Additionally, the relatively low encapsulation efficiency presents a challenge to achieving the requirement for sensitivity and stability in clinical diagnosis. In contrast, methods for the preparation of artificial lipid vesicles have been extensively developed.^[^ ^39^ ^]^ Depending on surface charge, liposomes can be categorized into cationic liposomes (CLs), neutral liposomes (NLs), or anionic liposomes (ALs).^[^ ^40^ ^]^

CLs are commonly used due to their efficient fusion of negatively charged EVs via electrostatic interactions,^[^ ^41^, ^42^, ^43^ ^]^ which enables signal amplification and readout through methods such as clustered regularly interspaced short palindromic repeats,^[^ ^44^ ^]^ molecular beacons,^[^ ^45^ ^]^ and DNA‐fueled molecular machines.^[^ ^46^ ^]^ Nevertheless, signal amplification probes are often composed of negatively charged nucleic acids,^[^ ^47^ ^]^ which may strongly interact with CLs, resulting in probe release challenges and compromised detection sensitivity. CLs have also been shown to adsorb certain irrelevant negatively charged biomolecules in body fluid,^[^ ^48^ ^]^ thereby affecting the efficiency of detection. Therefore, it is essential to develop a lipid vesicle detection system that combines high fusion efficiency, effective probe release, and the capacity for large‐scale preparation to ensure the high sensitivity and robustness required for clinical diagnosis.

**Scheme 1:**
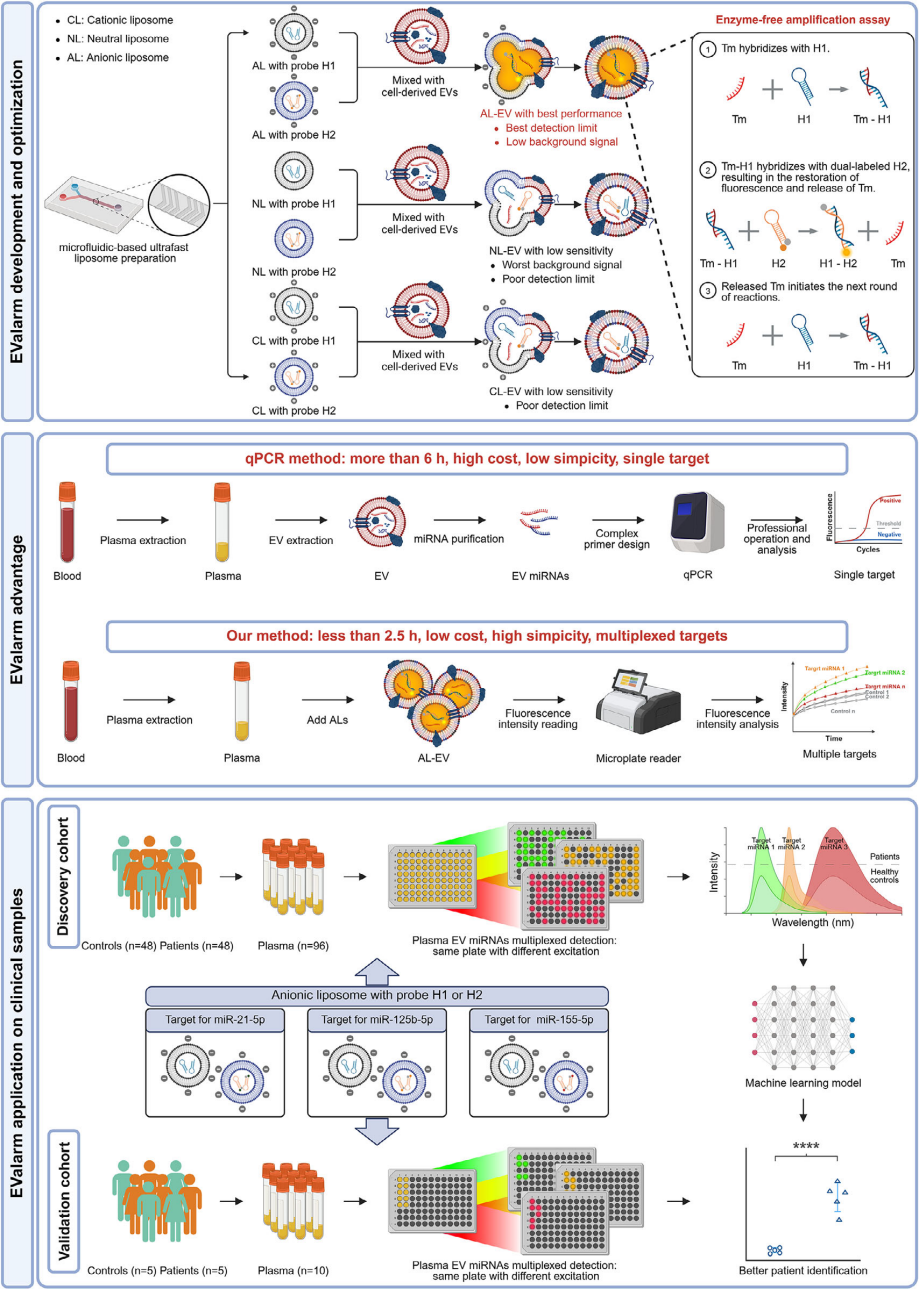
Development of EValarm, comparison with qPCR, and schematic workflow for direct multiplexed detection of plasma EV miRNAs. Systematic lipid screening identified ALs as the optimal carriers, exhibiting the best detection limits and suitability for chain displacement reaction and membrane fusion‐based assays. The detection system was efficiently synthesized using a microfluidic chip, enabling one‐step detection of multiplexed plasma EV miRNAs without complex pretreatment. The accuracy of detecting three EV miRNAs was validated in 48 malignant lymphoma samples and 48 HC. Combining an artificial intelligence model further enhanced discriminatory performance. An additional 5 patient samples and 5 HC demonstrated the high reliability of EValarm. (Tm: target miRNA, H1/H2, hairpin probes) Created with BioRender.com.

Here, a novel and efficient strategy, EValarm (Anionic Liposome Assisted miRNAs Monitoring for Extracellular Vesicles) was developed for simultaneous multiplexed detection of plasma EV miRNAs (**Scheme** **1**). A systematic evaluation of membrane fusion efficiency was conducted to optimize liposome surface charge. The results demonstrated that ALs serve as optimal nanocarriers for EV miRNA detection. Upon membrane fusion, target miRNAs (Tm) in EVs initiate a toehold‐mediated strand displacement reaction. The reaction catalyzes the assembly of hairpin DNA probes, resulting in signal amplification. Specifically, unlabeled hairpin probe H1 and dual‐labeled probe H2 (with a fluorophore and quencher) were encapsulated into liposomes using a microfluidic chip. The encapsulation efficiency of CLs, NLs, and ALs for hairpin probes was first evaluated. Then, the fusion kinetics of the three kinds of composite liposomes with EV were measured. Upon fusion, Tm catalyzed the formation of fluorescent H1/H2 products via a toehold‐mediated strand displacement reaction. We further optimized the reaction kinetics and fusion conditions for maximum sensitivity. Our results revealed distinct performance characteristics of the three liposomes. CLs demonstrated the lowest sensitivity, due to electrostatic interactions that hinder probe release and target recognition. NLs exhibited elevated background signals, thus limiting application. ALs, for the first time, were proven to offer lower background and superior limits of detection (LOD), representing the most favorable choice for membrane fusion‐based detection with the assistance of polyethylene glycol (PEG) and salt ions. Moreover, the novel membrane fusion strategy based on ALs demonstrated the same accuracy as the gold standard qPCR. Under optimized conditions, a simple incubation of preassembled reagents enabled direct, multiplexed detection of EV miRNAs in plasma samples from healthy controls (HC) (n = 48) and lymphoma patients (n = 48) with satisfactory accuracy in combination with an artificial intelligence model (AUC > 0.99). The reliability of EValarm was confirmed using an additional 10 samples from patients and HC. Through systematic evaluation of the surface charge characteristics of liposomes, we developed a novel anionic liposome‐assisted strategy, termed EValarm, for direct multiplexed detection of plasma EV miRNAs. More importantly, EValarm has been demonstrated to be user‐friendly, highly accurate, and suitable for batch production, thus supporting its strong potential for clinical application.

## 2 Results and Discussion

### 2.1 Synthesis of Composite Liposomes with Different Charges

To verify effective encapsulation of the hairpin probe in liposomes with various charges, we employed a microfluidic platform to synthesize probe‐loaded CLs, NLs, and ALs. By maintaining a constant lipid concentration in the organic phase and adjusting the flow rate ratio between the organic and aqueous phases, three types of composite liposomes were successfully generated. Dynamic light scattering (DLS) measurements confirmed the adequate dispersion and size stability of the prepared liposomes (Figures S1–S3, Supporting Information).

**Figure 1:**
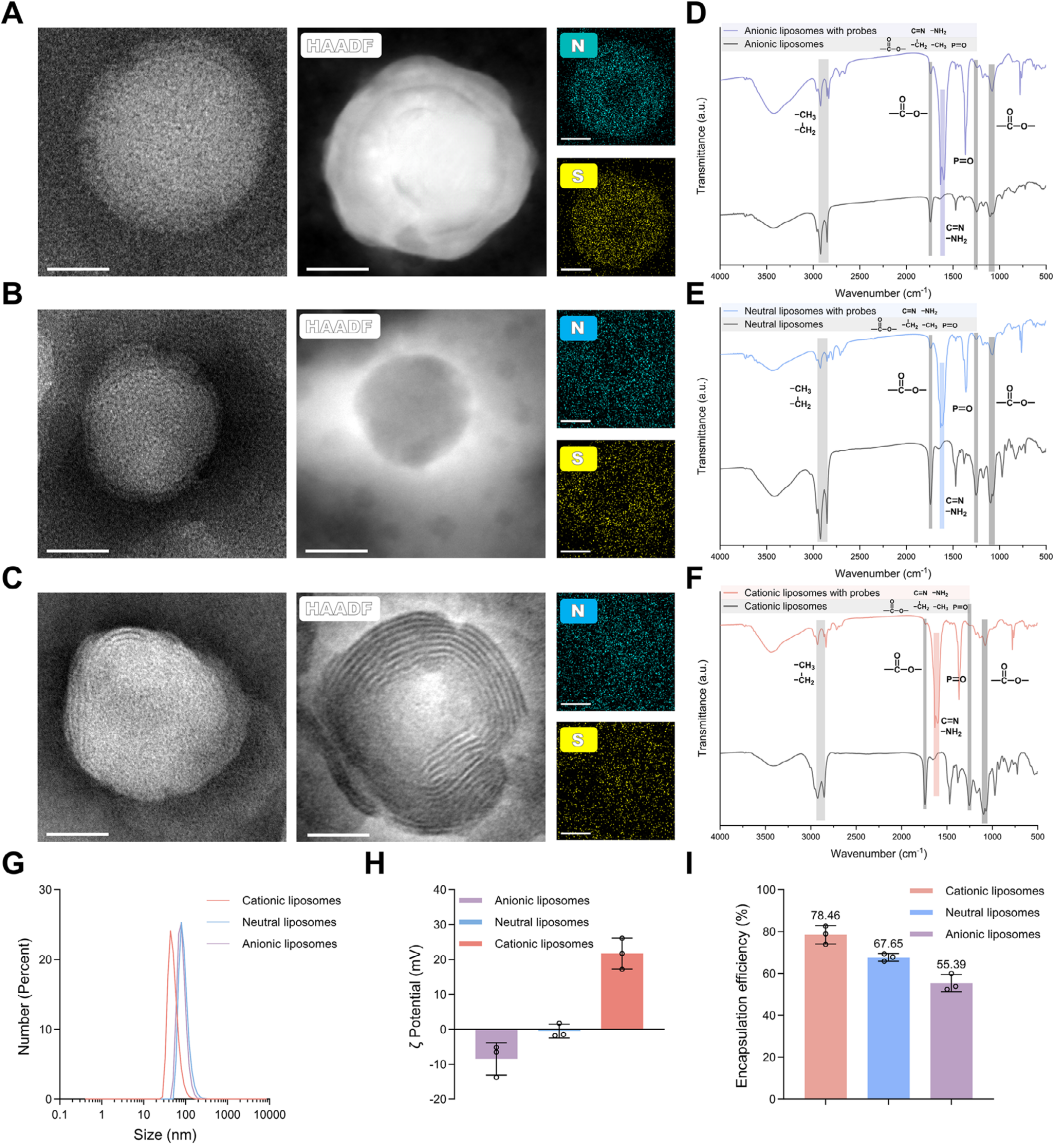
Characterization and probe encapsulation efficiency of composite liposomes with different charges. A–C), Representative TEM images, high‐angle annular dark field (HAADF) images, and elemental mapping showing encapsulated nucleic acid probes within anionic (A), neutral (B), and cationic (C) liposomes. Scale bars, 50 nm. D–F), FT‐IR confirming successful probe encapsulation in anionic (D), neutral (E), and cationic (F) liposomes. G), Hydrodynamic diameters of the composite liposomes. H), ζ potential measurements of the composite liposomes. I), Encapsulation efficiency of probes within the composite liposomes. Experiments were performed in triplicate. For (H) and (I), the data represent the mean ± SD (n = 3).

Hairpin probes were labeled with a sulfur‐containing fluorescent dye (Cy5, Table S1, Supporting Information). Transmission electron microscopy (TEM) revealed the characteristic spherical morphology of ALs (**Figure** **1**A), NLs (Figure 1B), and CLs (Figure 1C), confirming successful liposome formation. Elemental mapping showed spatial co‐localization of nitrogen (from lipid components) and sulfur (from the Cy5‐labeled probes), indicating successful probe loading into the liposomes (Figure 1A–C). These conclusions were supported by Fourier transform infrared spectroscopy (FT‐IR) (Figure 1D–F), where the absorption peaks at 1690–1630 cm^−1^ correspond to the C = N and amino group signals specific to hairpin probes.^[^ ^49^ ^]^ The hydrodynamic diameters of the liposomes were ≈92.4 nm (CLs), 113.3 nm (NLs), and 107.3 nm (ALs), consistent with TEM images (Figure 1G; Figures S1–S3, Supporting Information). Since DLS measures hydrated particle size while TEM measures actual particle size, DLS results were larger due to the widely reported influence of the hydration layer.^[^ ^50^, ^51^ ^]^ ζ potential measurements further confirmed the distinct surface charges of the three liposome types (Figure 1H). To quantify encapsulation efficiency, Cy5‐labeled hairpin probes (H1‐Cy5 and H2‐Cy5, Table S1, Supporting Information) were employed. Fluorescence intensity was measured before and after purification to remove free probes, and encapsulation efficiency was calculated based on Cy5 fluorescence signals (Figure 1I). As expected, CLs showed the highest encapsulation efficiency due to electrostatic attraction with the negatively charged probes. Conversely, ALs exhibited the lowest encapsulation efficiency due to electrostatic repulsion. However, whether higher encapsulation leads to improved detection performance remains to be determined in subsequent experiments.

### 2.2 Performance Evaluation of Target miRNA‐triggered Catalytic DNA Machinery

**Figure 2:**
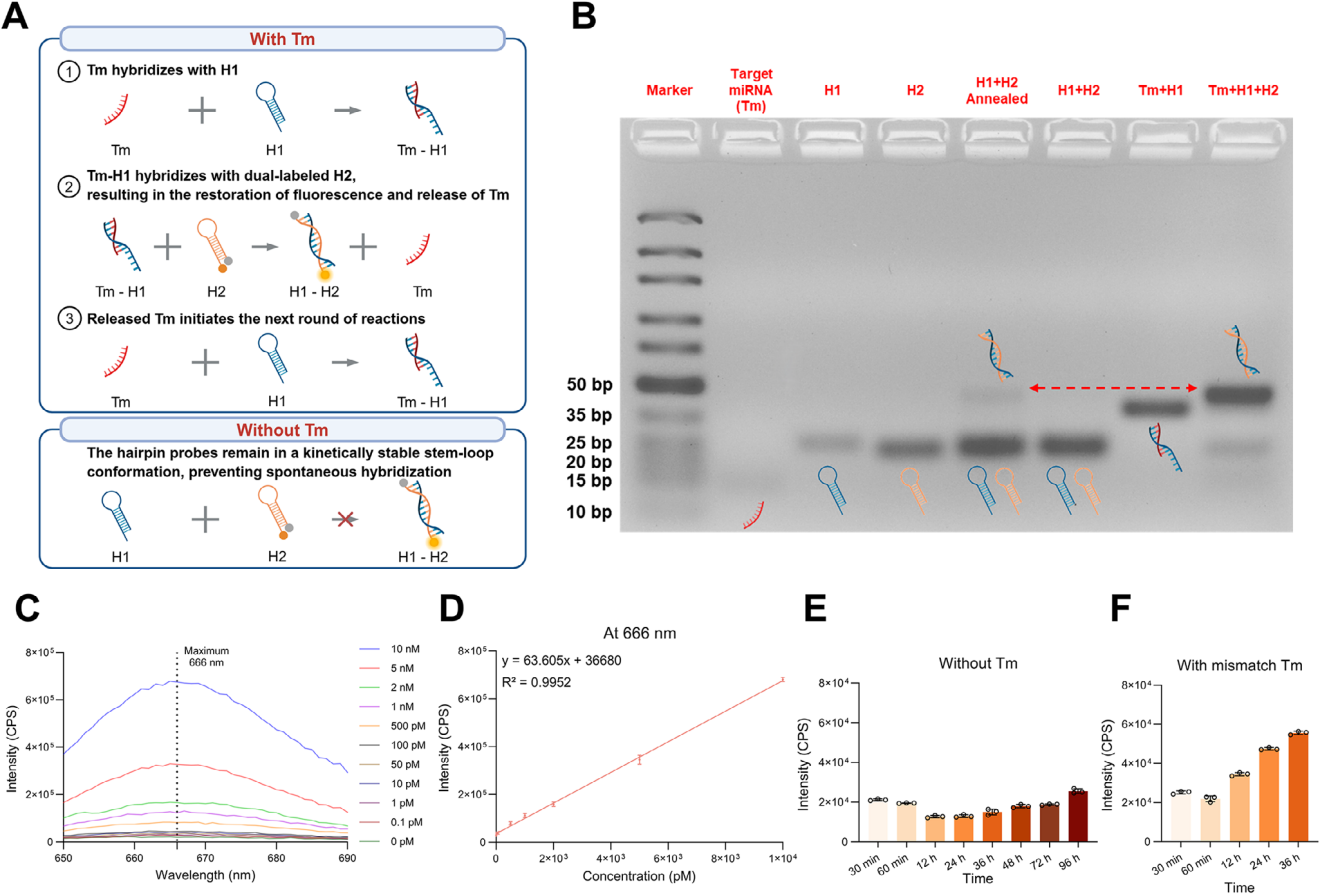
Toehold‐mediated strand displacement reaction enabling the detection of Tm. A), Schematic illustration of the strand displacement reaction triggered by Tm. Created with BioRender.com. B), Agarose gel electrophoresis analysis of different nucleic acid inputs. Lane 1: DNA ladder; Lanes 2–8: Tm, H1, H2, annealed H1+H2, H1+H2 (unannealed), Tm+H1, and Tm+H1+H2, respectively. C,D), Fluorescence emission spectra of the H1/H2 system with increasing concentrations of Tm (C) and the corresponding linear correlation between fluorescence intensity (at the maximum emission wavelength) and Tm concentration (D). E,F), Stability of the H1/H2 probe system in the absence of Tm (E) and in the presence of a mismatched miRNA sequence (F). Experiments were performed in triplicate. For (D), (E), and (F), the data represent the mean ± SD (n = 3).

To detect Tm after the membrane fusion process, a catalytic DNA machinery was designed. The system was based on a toehold‐mediated strand displacement reaction, achieving signal amplification through hairpin probes H1 and H2. Due to the proven overexpression in lymphoma cells (e.g., Raji cells), miR‐155‐5p, miR‐125b‐5p, and miR‐21‐5p were selected as Tm.^[^ ^52^, ^53^, ^54^ ^]^ In the absence of Tm, the hairpin probes remained in a kinetically stable stem‐loop conformation, thereby preventing spontaneous hybridization (Figure S4, Supporting Information). Upon the addition of miRNA targets such as miR‐155‐5p, Tm hybridized with H1, opening its loop and exposing a single‐stranded domain that initiated hybridization with H2. Tm was then released via a toehold‐mediated strand displacement, catalyzing further rounds of H1/H2 assembly and generating numerous duplex DNA products (**Figure** **2**A).

Agarose gel electrophoresis was performed with different nucleic acid inputs (Figure 2B). Tm (miR‐155‐5p), H1, and H2 exhibited their respective bands in Lanes 2 to 4. In Lane 5, annealed H1 and H2 generated a distinct band corresponding to the H1/H2 duplex. In contrast, no duplex formation was observed in Lane 6 (unannealed H1 and H2 without Tm), confirming the kinetic inhibition of spontaneous hybridization. Lane 7, which contained Tm and H1, exhibited a shifted band, indicating the formation of a Tm/H1 complex. In Lane 8, the combination of Tm, H1, and H2 reproduced the H1/H2 duplex band observed in Lane 5, thereby demonstrating the successful Tm‐triggered reaction. Similar results were obtained for miR‐125b‐5p and miR‐21‐5p (Figures S5 and S6, Supporting Information).

To assess the signal amplification behavior, a Cy5‐ and BHQ‐labeled H2 probe was developed (Figure 2C). Without Tm, the stem‐loop structure maintained proximity between Cy5 and BHQ, quenching the fluorescence. Upon H1 activation by Tm, H2 was opened via hybridization, leading to the formation of H1/H2 duplex. The spatial separation of Cy5 and BHQ resulted in the restoration of fluorescence. The reaction process reached nearly completion within 60 min (Figure S7, Supporting Information).^[^ ^55^ ^]^ Under optimal conditions, the limit of detection (LOD) for miR‐155‐5p was 50.3 fM (Figure 2D; Figure S8, Supporting Information). In the absence of Tm or with mismatched miRNA sequences, the fluorescence background remained suppressed (Figure 2E,F; Figure S7, Supporting Information). For miR‐125b‐5p and miR‐21‐5p, H2 probes were labeled with ROX/BHQ and FAM/BHQ, respectively. These systems similarly exhibited high sensitivity and specificity (miR‐125b‐5p: 0.016 fM, miR‐21‐5p: 72.2 fM) (Figures S5 and S6, Supporting Information).

### 2.3 Investigation of Liposome‐EV Membrane Fusion

**Figure 3:**
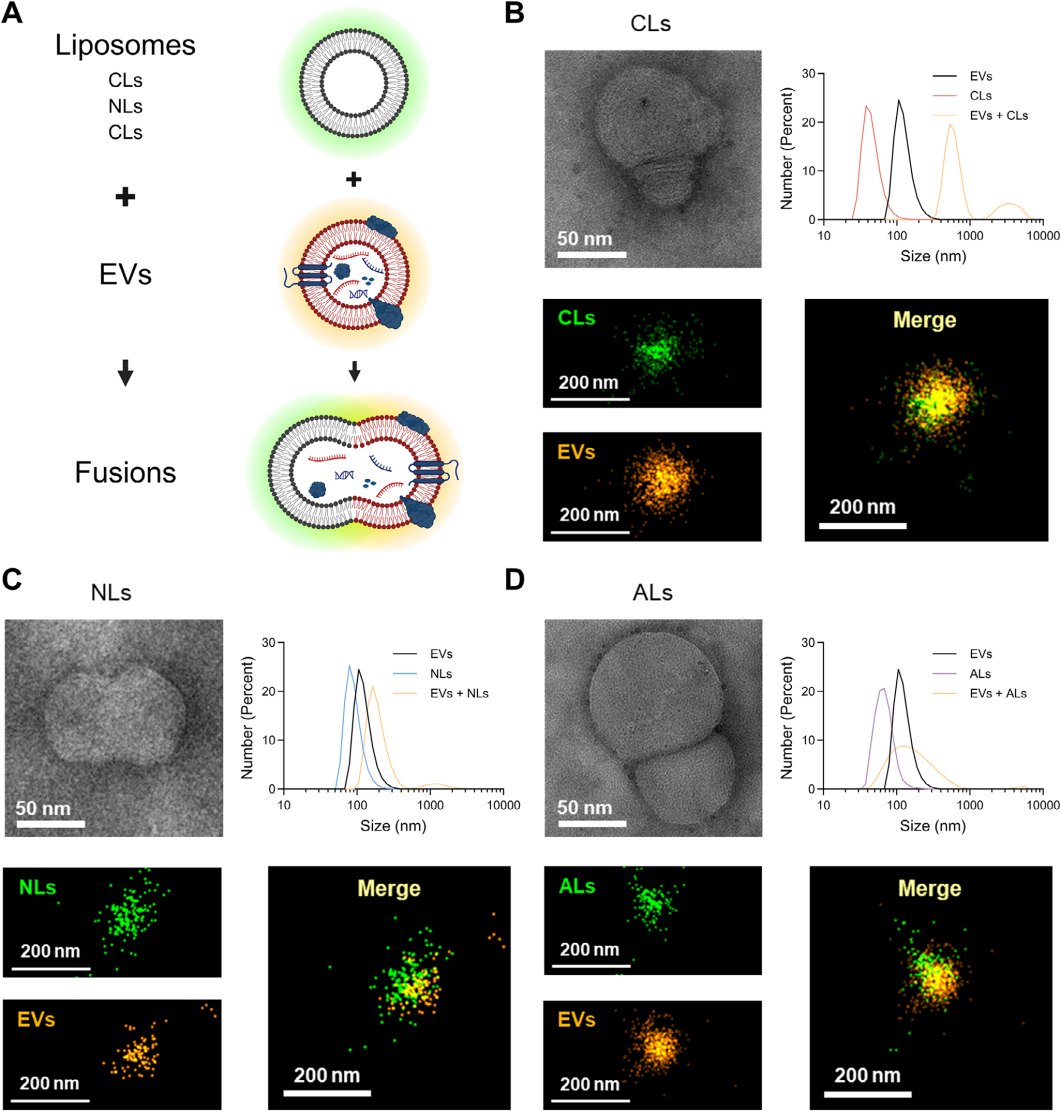
Characterization of membrane fusion feasibility between EVs and composite liposomes with different charges. A) Schematic illustration of the membrane fusion process. Created with BioRender.com. B–D), Representative characterization of membrane fusion between CLs (B), NLs (C), and ALs (D) with EVs, including TEM images, hydrodynamic size changes, and super‐resolution microscope images. Liposomes were labeled with membrane dyes (green, Ex: 488 nm), and EVs were labeled with tetraspanin trio detection (CD9, CD63, and CD81, orange, Ex: 561 nm).

To assess the feasibility of membrane fusion, liposomes and tumor‐derived EVs were labeled with membrane dyes and tetraspanin trio detection, respectively, and incubated in PBS buffer containing PEG to promote fusion,^[^ ^30^ ^]^ followed by characterization using TEM, DLS, and super‐resolution microscopy (**Figure** **3**A). Raji cells‐derived EVs were extracted by ultracentrifugation (UC) and characterized by nanoparticle tracking analysis (NTA), western blot, and ζ potential measurements (Figure S9, Supporting Information). The characterization results were consistent with previous reports,^[^ ^41^ ^]^ indicating the successful extraction of EVs. The preparation of composite liposomes followed the previously described protocols. Fusion between CLs, NLs, ALs, and EVs was then observed by TEM, DLS, and super‐resolution microscopy (Figure 3B–D). The fusion vesicles were clearly observed in TEM images, and changes in hydrodynamic diameter further confirmed the occurrence of membrane fusion. Super‐resolution analysis of EVs and the three liposomes was performed using direct stochastic optical reconstruction microscopy (dSTORM). Based on the stochastic blinking of individual fluorophores, dSTORM enables the observation of nanoscale particles by acquiring a large number of images, allowing for the visualization of fusion events within fluorescence fields. As demonstrated by super‐resolution microscope images, liposomes labeled with membrane dyes (green pseudocolor) and EVs labeled with tetraspanin trio detection (orange pseudocolor) exhibit significant fluorescence overlap. These results confirm that all three liposomes are capable of fusion with EVs. The fusion efficiency of the three liposomes with EVs was further determined using fluorescence resonance energy transfer (Figure S10, Supporting Information). The results showed that CLs exhibited the highest efficiency compared to NLs and ALs.

**Figure 4:**
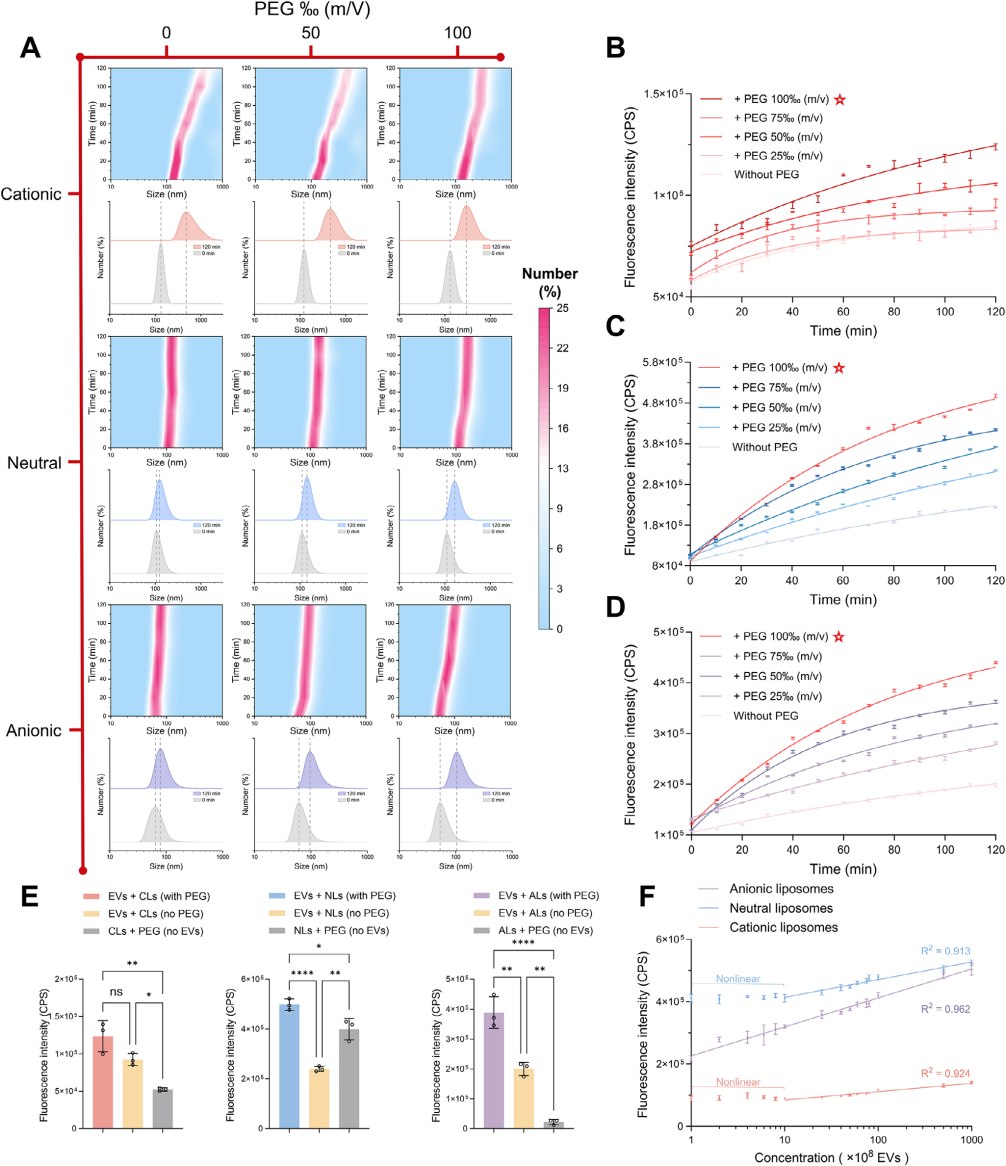
Identification of the optimal composite liposome. A) In situ hydrodynamic size profiles showing the membrane fusion kinetics between EVs derived from Raji cells and CLs, NLs, and ALs under various PEG concentrations. B–D), Fluorescence kinetics of CLs (B), NLs (C), and ALs (D) in the presence of EVs under different PEG concentrations. E) Comparative fluorescence intensities of CLs, NLs, and ALs in the absence of EVs and PEG. F) Linear correlation between fluorescence intensity and EV concentration for CLs, NLs, and ALs. For (B), (C), (D), (E), and (F), the data are presented as mean ± SD from three independent experiments. ^*^ *p* < 0.05, ^**^ *p* < 0.01, ^****^ *p* < 0.0001. ns, no significant difference.

As previously reported, PEG facilitates dehydration of adjacent membrane surfaces, promoting lipid rearrangement and subsequent membrane integration and content mixing.^[^ ^56^ ^]^ Additionally, the salt concentration in the reaction system was modified, as salts have been demonstrated to reduce electrostatic repulsion between lipids.^[^ ^57^, ^58^ ^]^ This principle has been widely applied in nucleic acid extraction processes to overcome repulsion with silica beads.^[^ ^59^, ^60^ ^]^ The fusion kinetics were investigated by monitoring in situ particle size changes using DLS (**Figure** **4**A). For CLs, fusion with EVs was suppressed at 100‰ PEG concentration (m/V) due to steric hindrance, which offset the electrostatic attraction by preventing direct contact of lipid headgroups. In contrast, NLs and ALs exhibited minimal fusion with EVs without PEG, while PEG‐induced dehydration significantly reduced the hydration layer thickness and promoted fusion.^[^ ^34^, ^56^ ^]^ Fusion rates of NLs and ALs were positively correlated with PEG concentration up to 100‰. In the absence of PEG, an increase in salt concentration resulted in a minimal effect on fusion (Figure S11, Supporting Information). Therefore, the impact of salt concentration was further examined in a 50‰ PEG buffer (Figure S12, Supporting Information). In the case of CLs, the effect of salt was negligible due to their robust electrostatic interaction with EVs.^[^ ^61^ ^]^ For NLs, salt ions promoted membrane contact and fusion by reducing hydration repulsion with EVs. For negatively charged ALs and EVs, salt reduced electrostatic repulsion and shielded EV surface charges, enabling further fusion.

In order to study the kinetics of membrane fusion detection, H2 probes labeled with both Cy5 and BHQ were applied (Figure 4B–D). The H1 and H2 probes were encapsulated in liposomes and stored separately. For detecting EV miRNAs, we performed additional super‐resolution analysis to evaluate the fusion behavior of probe‐loaded liposomes. As demonstrated by super‐resolution microscope images, Cy5‐labeled probes (magenta pseudocolor), liposomes labeled with membrane dyes (green pseudocolor), and EVs labeled with tetraspanin trio detection (orange pseudocolor) exhibit significant fluorescence overlap (Figure S13, Supporting Information), confirming the successful fusion between probe‐loaded liposomes and EVs. For all kinds of composite liposomes, increasing PEG concentration enhanced fluorescence signals, indicating fusion and probe activation. PEG also provided volume exclusion effects, concentrating nucleic acids, shortening hybridization time, and promoting toehold binding and strand displacement.^[^ ^62^, ^63^, ^64^ ^]^ However, excessive PEG caused content leakage and increased background fluorescence (Figure S14, Supporting Information).^[^ ^65^ ^]^ Therefore, 100‰ PEG was considered the optimal concentration. For consistency, changes in salt ion concentration were also performed within the same buffer system as described previously (Figure S15, Supporting Information). All three types of liposomes exhibited an optimal salt concentration range during membrane fusion detection, as evidenced by a bell‐shaped response curve. This can be attributed to the ability of moderate salt levels to reduce electrostatic repulsion and enhance toehold hybridization,^[^ ^66^ ^]^ whereas excessively high salt concentrations stabilize the double‐helix structure and suppress target dissociation, thereby slowing the reaction kinetics.^[^ ^67^ ^]^

Under optimal conditions, the detection performance of the three kinds of liposomes was evaluated (Figure 4E). CLs performed with the lowest sensitivity due to the difficulty of probe release from cationic membranes,^[^ ^68^ ^]^ which in turn affected the contact with Tm and the triggering of the catalytic assembly. NLs were observed to have improved detection efficiency with increased background signal intensity, likely due to the presence of spontaneous fusion, which induces non‐specific binding of probes, potentially affecting the detection limit. In contrast, ALs demonstrated lower background and enhanced signal response, making it promising as a carrier for EV detection. Although CLs show strong electrostatic attraction to negatively charged EVs,^[^ ^69^ ^]^ which contributes to their high fusion efficiency, their positive surface also causes nonspecific adsorption of nucleic acids, including DNA probes and Tm. This electrostatic trapping limits probe mobility and reduces hybridization efficiency. NLs exhibit elevated background signals, thereby reducing detection sensitivity. In contrast, ALs do not adsorb nucleic acids, allowing probes to remain accessible and enabling efficient toehold‐mediated strand displacement. A notable finding is that CLs with the highest fusion efficiency exhibit the poorest detection performance. This indicates that fusion efficiency alone does not determine detection capability, while probe accessibility proves more critical for fusion detection, highlighting the superiority of ALs. Moreover, PEG‐induced dehydration and salt‐induced charge shielding act together to overcome both hydration and electrostatic barriers between membranes. These combined effects explain the superior fusion efficiency and reproducibility of AL‐based detection.

Finally, the LOD of CLs, NLs, and ALs was determined under optimal conditions. EVs were purified and diluted to different concentrations, and then incubated with each kind of liposome for 120 min. All three composite liposomes performed with a good linear range. ALs demonstrated the lowest LOD (particles/mL, ALs: 6.1 × 10^7^, NLs: 1.7 × 10^9^, CLs: 4 × 10^10^, Table S2, Supporting Information) and a wider linear range (Figure 4F) compared to the other two liposomes, indicating their superior performance for EV miRNA detection through membrane fusion. The inconsistency between fusion efficiency and detection performance was most evident for CLs, which showed maximal fusion yet minimal detectability, highlighting the critical role of probe accessibility in the assay. These results demonstrate that ALs are the superior probe carriers with relatively excellent performance in membrane fusion detection of EV miRNAs.

### 2.4 Validation of Detection System Reliability

**Figure 5:**
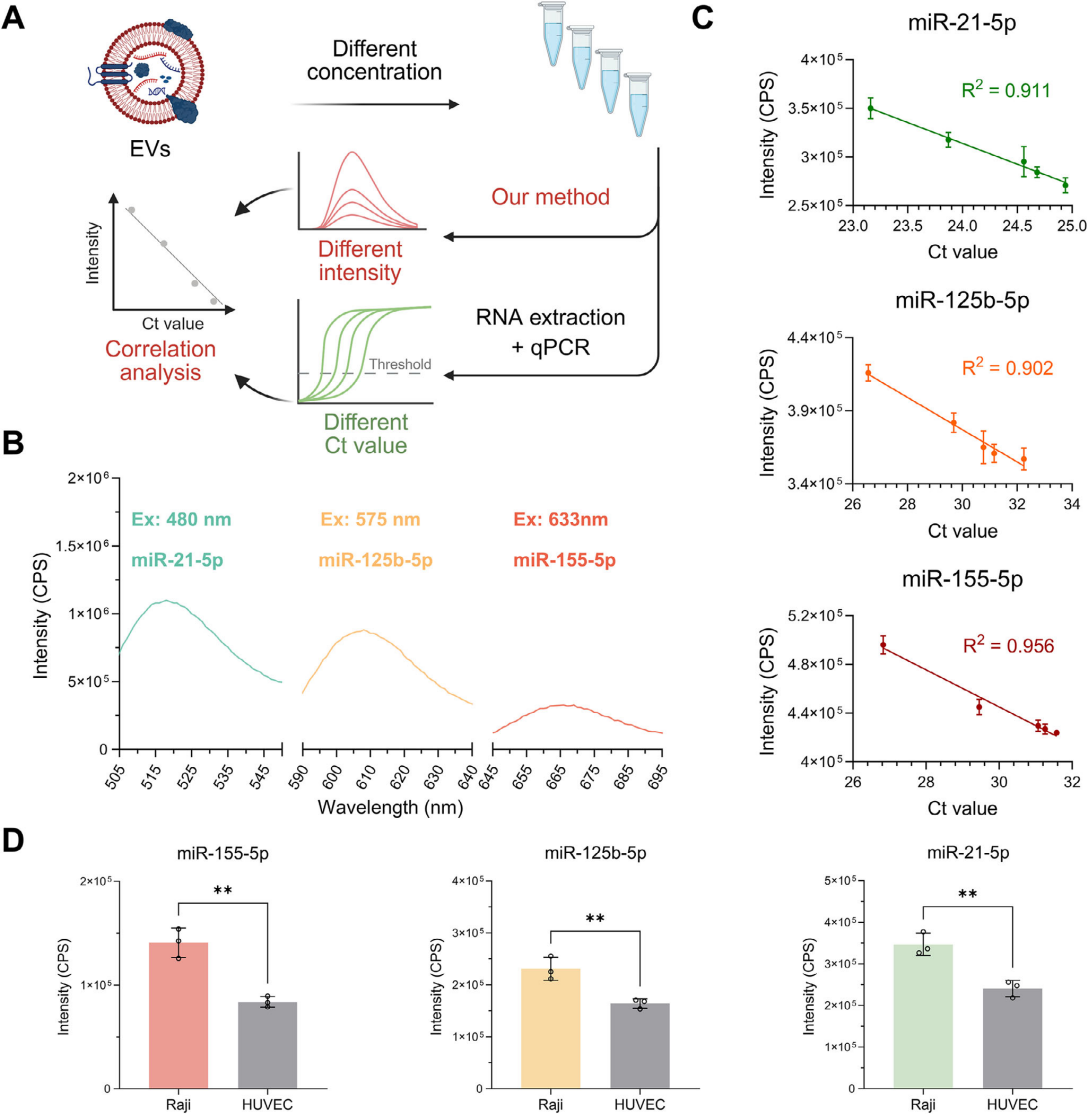
Multiplexed detection of miRNAs in EVs at varying concentrations and correlation with qPCR. A) Schematic illustration of the membrane fusion‐based strategy and qPCR for detecting miR‐21‐5p, miR‐125b‐5p, and miR‐155‐5p in EVs at different concentrations. Created with BioRender.com. B) Simultaneous detection of three miRNAs within EVs in a single reaction system. The H2 probe for miR‐21‐5p was labeled with FAM (Ex: 480 nm, Em: 520 nm) and BHQ1; the probe for miR‐125b‐5p with ROX (Ex: 575 nm, Em: 615 nm) and BHQ2; and the probe for miR‐155‐5p with Cy5 (Ex: 633 nm, Em: 670 nm) and BHQ3. C) Correlation between Ct values from qPCR and fluorescence intensities from the membrane fusion‐based assay across various EV concentrations. D) In situ detection of miR‐21‐5p, miR‐125b‐5p, and miR‐155‐5p expression level in EVs derived from Raji cells and HUVEC. ^**^ *p* < 0.01. For (C) and (D), the data represent the mean ± SD (n = 3).

To verify the effectiveness of our proposed detection method, we performed a comparative analysis with the current gold standard qPCR. EVs derived from Raji cells were prepared at different concentrations, and the corresponding cycle threshold (Ct) values were obtained by qPCR. In parallel, under optimized reaction conditions, our method was used to detect fluorescence intensity at the endpoint of the reaction for each EV concentration, which was then compared with the corresponding Ct values (**Figure** **5**A). As previously described, miR‐21‐5p, miR‐125b‐5p, and miR‐155‐5p were selected as targets due to their potential as biomarkers. Three targets were tested simultaneously, with three different groups of H1 and H2 probes. In the samples with the highest EV concentration, the fusion reaction successfully triggered three distinct fluorescence emission peaks (Figure 5B), demonstrating the feasibility of multiplexed detection. Fluorescence intensities at the corresponding maximum emission wavelengths were recorded for EVs at different concentrations and compared to their respective qPCR Ct values (Figure 5C). The results showed strong correlations between our method and qPCR for all three Tm (R^2^ = 0.911 for miR‐21‐5p, 0.902 for miR‐125b‐5p, and 0.956 for miR‐155‐5p). To further evaluate the reliability of our method, EVs derived from human umbilical vein endothelial cells (HUVEC) were analyzed. The relative expression differences of the three miRNAs between HUVEC‐ and Raji‐derived EVs were measured by qPCR (Figure S16, Supporting Information). Notably, the same results as the gold standard further demonstrated the accuracy of our proposed method (Figure 5D).

### 2.5 Performance Characterization in the Identification of Malignant Lymphoma Patients

**Figure 6:**
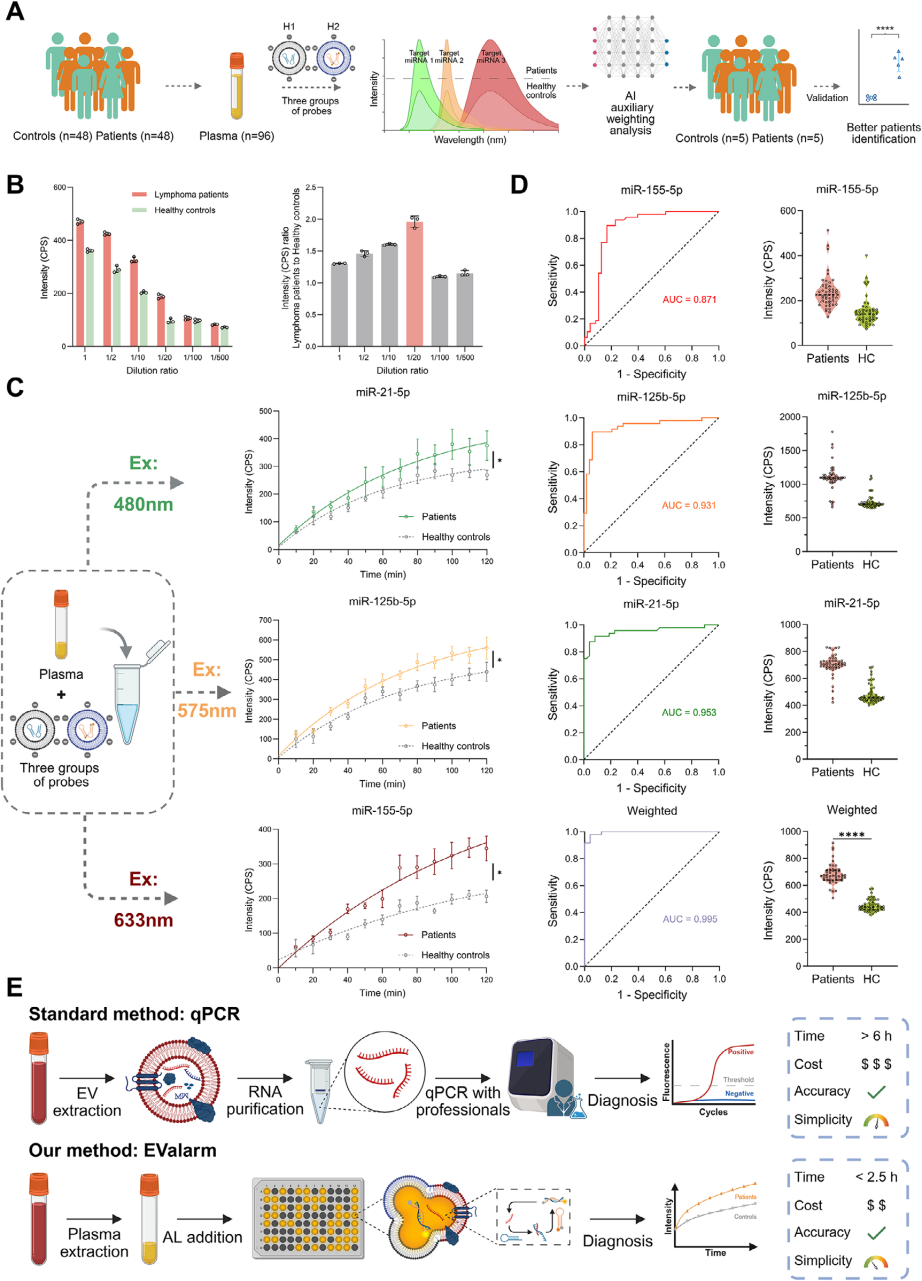
Direct detection of EV miRNAs in plasma from lymphoma patients and HC. A) Schematic illustration of EValarm for distinguishing lymphoma patients from HC by directly detecting miR‐21‐5p, miR‐125b‐5p, and miR‐155‐5p in plasma‐derived EVs. B) Optimization of plasma dilution ratios for optimal detection performance. The optimal dilution was defined as the condition yielding the highest fluorescence intensity ratio between lymphoma and control samples after a 120‐min reaction. C) Fluorescence intensities after 120 min in plasma samples from lymphoma patients and HC. D), ROC and scatter plots of direct detection of the three EV miRNAs in plasma from 48 lymphoma patients and 48 HC; ROC analysis before and after applying an artificial intelligence algorithm to weight the three miRNAs. E) Comparison of our methods with conventional qPCR in clinical sample detection. Created with BioRender.com. The data in (C) represent the mean ± SD (n = 3). ^*^ *p* < 0.05, ^****^ *p* < 0.0001.

We further evaluated the performance of EValarm by analyzing plasma samples from HC and patients with non‐Hodgkin's lymphoma (NHL) (**Figure** **6**A). To validate the effectiveness of the indicators, EVs were isolated from both groups by UC and characterized (Figure S17, Supporting Information), strictly following the methodology reported by previous studies.^[^ ^26^, ^41^ ^]^ Then, gold standard qPCR analysis confirmed upregulated expression levels of miR‐21‐5p, miR‐125b‐5p, and miR‐155‐5p in EVs from plasma of NHL patients (Figure S18, Supporting Information). Before analysis by EValarm, plasma samples from both groups were diluted at different ratios to determine the optimal concentration for detection. Fluorescence intensity at the endpoint of the reaction was measured (Tm: miR‐155‐5p), and the fluorescence ratios between the lymphoma patients and HC were analyzed to determine that the optimal dilution condition was 1:20 (Figure 6B). Samples from patients showed significantly higher fluorescence signals compared to those from HC (*p* < 0.05) (Figure 6C), consistent with the qPCR results (Figure S18, Supporting Information), which demonstrated that our proposed assay achieved the same accuracy as the gold standard for multiplexed detection.

Subsequently, plasma from 48 NHL patients and 48 HC were directly tested for multiplexed targets simultaneously (Figure 6D). Parallel detection of miR‐155‐5p, miR‐125b‐5p, and miR‐21‐5p showed good accuracy. The receiver operating characteristic curve (ROC) revealed robust discrimination between patients and HC, with the area under the curve (AUC) all greater than 0.85. To further enhance detection accuracy, an artificial intelligence algorithm was employed to integrate the fluorescence signals of the three miRNAs. Using a machine learning model, optimal weights were computed for each signal (miR‐155‐5p: 0.3655, miR‐125b‐5p: 0.3612, miR‐21‐5p: 0.2733), which were then linearly combined to generate a final adjusted output value (Table S3, Supporting Information), resulting in significantly improved diagnostic accuracy (*p* < 0.0001, AUC = 0.995). Additional validation using 10 independent clinical samples confirmed the strong discrimination of the adjusted indicator (Table S4, Supporting Information). Among the 10 samples, 3 samples were misclassified based on a single indicator. In contrast, only 1 sample was incorrectly identified after weight adjustment based on three indicators. These findings validate the capability of EValarm in distinguishing lymphoma patients from HC and suggest its broader potential in cancer diagnostics. Under optimized lipid composition and reaction conditions, EValarm enables the simultaneous detection of multiplexed EV miRNA within a single reaction system, effectively improving efficiency and reducing cost, thus emphasizing the practical potential of membrane fusion strategies combined with enzyme‐free amplification for clinical applications.

In our study, microfluidic chips were employed to prepare probe‐loaded liposomes (H1 and H2). Fluorescent dyes and quenchers were incorporated into H2 to enable multiplexed miRNA detection, offering an alternative to conventional qPCR. By monitoring particle size and fluorescence changes in real time, we optimized the liposome charge characteristics and discovered that ALs are most suitable for membrane fusion‐based detection. Once the ALs are prepared, rapid and parallel multi‐target detection of clinical samples can be performed, greatly simplifying the workflow and reducing testing duration. Moreover, our results validate that miR‐155‐5p, miR‐125b‐5p, and miR‐21‐5p can distinguish between malignant lymphoma patients and HC, and these biomarkers hold significant value for process monitoring and prognosis evaluation in malignant lymphoma. To our knowledge, this is the first one‐step method for directly detecting multiplexed EV miRNAs in plasma from lymphoma patients. Moreover, the composite liposomes demonstrated long‐term stability over 14 days, making them suitable for preproduction and storage. The integration of artificial intelligence algorithms further improved detection accuracy, ensuring the ability of EValarm to accurately discriminate between lymphoma patients and HC.

Although the expression levels of EV miRNAs are critical for monitoring the progression of malignant lymphoma, a simple, rapid, and clinically applicable detection method remains to be established. While qPCR remains the gold standard for nucleic acid detection due to its high sensitivity and specificity, its application in plasma EVs analysis is limited by the need for complex pre‐processing steps such as EV isolation and nucleic acid extraction. Moreover, it relies on enzyme‐based amplification, which makes the procedure more laborious and instrument‐dependent (Figure 6E). In contrast, our proposed method, EValarm, offers a sensitive, rapid, and enzyme‐free alternative with more gentle reaction conditions. Comparative analysis with several representative EV miRNA detection systems demonstrates that EValarm achieves comparable detection performance while maintaining lower system complexity and offering greater scalability (Figure S19, Supporting Information). Our method allows direct analysis of plasma samples for multiplexed EV miRNAs, with great potential for cancer diagnosis and prognosis monitoring.

## 3 Conclusion

EV miRNAs have emerged as promising biomarkers for liquid biopsy, owing to their stability in body fluids and ability to reflect the pathological state of their parental cells. Notably, EV miRNAs such as miR‐155‐5p, miR‐125b‐5p, and miR‐21‐5p have been shown to play important roles in monitoring the progression, therapeutic response, and prognosis of malignant lymphomas, highlighting their clinical relevance as biomarkers. However, current detection approaches typically require EV isolation and RNA extraction, which are labor‐intensive, time‐consuming, and lead to sample loss, ultimately compromising sensitivity and limiting clinical translation. Membrane fusion‐based detection offers a potential solution. Natural CMVs have been shown to specifically recognize EVs through homotypic fusion, thereby enabling in situ analysis of miRNAs.^[^ ^37^ ^]^ However, the preparation of CMVs is complex, difficult to scale up, and limited by low probe encapsulation efficiency. The synthesis of artificial liposomes is a relatively well‐developed process. However, CLs, the most prevalent type, exhibit strong interactions with nucleic acid probes and negatively charged biomolecules in biofluids, thereby restricting probe release and reducing sensitivity. Consequently, the development of a liposome‐based detection platform that integrates efficient fusion, effective probe release, and scalable preparation is imperative to advance EV miRNAs analysis toward clinical application.

In this study, we developed a novel platform, termed EValarm, based on AL‐assisted membrane fusion and catalytic DNA machinery that enables one‐step, direct, and multiplexed detection of EV miRNA in plasma, while minimizing the need for laborious sample pretreatment and associated miRNA loss. Following efficient membrane fusion, the Tm inside the EVs function as endogenous triggers to initiate the catalytic assembly of probes, thereby achieving signal amplification. After confirming ALs as the optimal delivery carrier, we demonstrated the detection capability of miR‐155‐5p with EVs derived from Raji cells. The method demonstrated a favorable limit of detection and a broad linear range, with the same accuracy as the gold standard qPCR. Furthermore, we validated our method using clinical plasma samples, targeting miR‐155‐5p, miR‐125b‐5p, and miR‐21‐5p as representative biomarkers. The results showed excellent performance in distinguishing lymphoma patients from HC, with the AUC all greater than 0.85. Through the integration of artificial intelligence algorithms for the analysis of the fluorescence signals of multiple indicators, we significantly improved the accuracy of the assay and achieved high diagnostic reliability (AUC > 0.99). The accuracy was further validated using an additional 10 clinical samples. The EValarm platform is simple, efficient, and user‐friendly, and can be readily adapted for detecting diverse EV miRNAs by modifying the DNA probes designed for toehold‐mediated strand displacement reactions. The composite ALs can be pre‐manufactured and stably stored for at least two weeks, requiring only a simple mixing step with the reaction solution prior to use, thereby streamlining the overall workflow. The clinical workflow enables fluorescence readout within 120 min from plasma samples through direct reaction with probe‐loaded ALs. Future efforts will focus on rapid and scalable production of composite liposomes, as well as further refinement of probe design and reaction optimization to extend the linear detection range and improve sensitivity. We have demonstrated the robust detection of three key miRNAs in plasma EVs, and future work will include expanding the detection of additional biomarkers and their application in lymphoma prognosis monitoring.

Notably, the expression levels of EV miRNAs have been well recognized as critical indicators for tracking the onset and progression of malignant lymphoma.^[^ ^4^ ^]^ Elevated expression of miR‐155‐5p has been associated with shorter progression‐free survival and is involved in immune evasion via PD‐L1 regulation.^[^ ^18^, ^70^, ^71^, ^72^, ^73^ ^]^ Upregulation of miR‐125b‐5p correlates with poor prognosis and modulates CD20 expression through the TNFAIP3 pathway,^[^ ^19^, ^74^ ^]^ making it a potential predictor of targeted therapy response.^[^ ^75^, ^76^ ^]^ As a well‐recognized indicator, miR‐21‐5p is closely associated with the regulation of multiple crucial signaling pathways.^[^ ^20^, ^77^ ^]^ It has not only been demonstrated to be useful for evaluating treatment response,^[^ ^78^ ^]^ but it is also associated with poor prognosis and increased tumor angiogenesis.^[^ ^79^ ^]^ Overall, EV miR‐155‐5p, miR‐125b‐5p, and miR‐21‐5p play important roles in prognosis evaluation, highlighting the potential clinical application of EValarm. However, the demonstration of distinct discriminatory capability among lymphoma subtypes has yet to be observed.^[^ ^70^, ^77^, ^80^, ^81^ ^]^ In future studies, further exploration of biomarker combinations holds significant importance for achieving more precise lymphoma classification and prognostic prediction. The EValarm platform enables the detection of multiplexed EV miRNAs, such as miR‐21‐5p. However, miR‐21‐5p has been implicated in various cancers, including ovarian, colorectal, and prostate cancers.^[^ ^31^, ^82^, ^83^ ^]^ MiR‐155‐5p has also been demonstrated to be upregulated in liver diseases.^[^ ^84^ ^]^ Given their broad expression across diverse tumor types, future studies will focus on integrating more representative miRNA panels to enhance tumor‐type specificity.

Accordingly, we present the first one‐step method for directly detecting all three EV miRNAs. While the mechanistic relationships between these miRNAs and their associated signaling pathways require further investigation, regular follow‐up and monitoring of these biomarkers in lymphoma patients will enhance our understanding of their clinical significance. Overall, we believe that our novel AL‐based membrane fusion strategy has significant potential for cancer detection and prognostic monitoring in clinical applications.

## 4 Experimental Section

### Gel Electrophoretic Analysis of Toehold‐Mediated Chain Displacement Reaction

Probes H1 and H2 were pre‐analyzed using NUPACK to simulate their structures.^[^ ^85^ ^]^ Different nucleic acid samples (Tm, H1, H2, H1 incubated with Tm, annealed duplex of H1 and H2, H1 and H2 without annealing, Tm incubated with H1 and H2) were prepared at a final concentration of 1 µM. After preparation, samples were mixed with 6 × TriTrack DNA loading buffer (Thermo Fisher Scientific) and were loaded onto a 5% agarose gel. The electrophoretic separation process was run in 1 × TAE buffer at 120 V for 40 min. Images of the resulting gels were obtained using an ImageQuant LAS 500 (GE Healthcare) system.

### Performance of Toehold‐Mediated Chain Displacement Reaction

Different target miRNAs (Tm), H1, and H2 probes for different Tm were dissolved with 1 × PBS buffer. Among them, H1 and H2 probes were diluted to a final concentration of 20 nM. Different concentrations of Tm were incubated with the probes at 37 °C, and the fluorescence intensity was measured at the end of the reaction.

### Synthesis of Three Different Charge Liposomes by Microfluidic Methods

The organic and aqueous phases introduced into the microfluidic chip were prepared separately. Hairpin probes H1 and H2 for different Tm were dissolved and pre‐treated, respectively. Hairpin probes were then diluted in 1 × PBS buffer to a final concentration of 200 nM. Lipid materials were separately dissolved in ethanol. The cationic liposomes (CLs) consisted of DOTMA, DMPC, and cholesterol in a molar ratio of 3:3:2. The neutral liposomes (NLs) were made from DMPC and cholesterol in a molar ratio of 3:1. The anionic liposomes (ALs) were prepared from DPPG, DMPC, and cholesterol in a molar ratio of 3:3:2. The total concentration of lipids in the organic phase was set at 1 mg mL^−1^ at the time of loading into the microfluidic chip. Cationic liposomes, neutral liposomes, and anionic liposomes were prepared at different flow rate ratios to explore the optimal conditions. All liposomes were purified to remove ethanol and were stored at 4 °C for further use.

### Characterization of Liposomes with Three Different Charges

Transmission electron microscopy (TEM), Fourier transform infrared spectroscopy (FT‐IR), hydrodynamic size, and ζ potential were used for liposome characteristics. A fluorescence spectrometer was used to calculate the probe encapsulation rate. The encapsulation rate was calculated using the following Equation 1.

(1) $$\left(F_{\mathrm{purified}}\;/\;F_{0}\right)\;\times \;100\%$$

*F_0_* is defined as the amount of probe added to the reaction system. *F_purified_* is defined as the amount of probe in the purified system.

### Cell Culture and Isolation of Extracellular Vesicles (EVs)

Raji cells and human umbilical vein endothelial cells (HUVEC) were grown in RPMI‐1640 with the addition of 10% FBS and 1% penicillin‐streptomycin antibiotic mixture in a humidified atmosphere containing 5% CO_2_ at 37 °C. Cell‐derived extracellular vesicles (EVs) were isolated from the supernatant of culture medium via ultracentrifugation (UC). Cells were separated from the culture medium once the cell confluence reached 80%–90%, and were then transferred to serum‐free medium (containing 1% antibiotic mixture) to be cultured at 37 °C for 48 h. EVs were then isolated by UC. The collected cell culture medium was sequentially centrifuged at 300 g for 10 min, 2000 g for 15 min, and 20 000 g for 60 min at 4 °C to remove intact cells and cellular debris. Subsequently, the supernatant was centrifuged at 140 000 g for 90 min at 4 °C twice. The obtained EVs were finally resuspended in PBS and stored at −80 °C for further use. For qPCR testing, samples were diluted to the same concentration. Measurement of the EV particle size and concentration was conducted on ZetaView (Particle Metrix), and ζ potential was conducted on Zetasizer Nano ZS90 (Malvern Panalytical).

### Transmission Electron Microscopy (TEM)

EVs purified by UC and different liposomes were dispersed in 1 × PBS buffer and diluted to appropriate concentrations. Subsequently, samples were prepared using negative staining with phosphotungstic acid and used for TEM observation (Talos F200X).

### Nanoparticle Tracking Analysis (NTA)

Cell‐derived EVs and plasma‐derived EVs purified by UC were dispersed in 1 × PBS buffer and diluted to a concentration suitable for NTA. ZetaView (Particle Metrix) was used to carry out the concentration and size distribution of EVs after calibration with 100 nm polystyrene particles. The instrumental parameters were set as brightness to 20, sensitivity to 70, and shutter to 70.

### Hydrodynamic Size and ζ Potential Characterization

EVs purified by UC and liposomes with three different charges were separately dispersed in 1 × PBS. Measurements of hydrodynamic size and ζ potential were conducted by dynamic light scattering (DLS) using a Zetasizer Nano ZS90 (Malvern Panalytical). The reported values represent the average of three independent replicates.

### Investigation of the Fusion Process of Liposomes with EVs

The fusion process between three different types of liposomes and EVs was investigated using super‐resolution microscopy, DLS analysis, and TEM. Super‐resolution analysis of EVs and the three liposomes was performed using direct stochastic optical reconstruction microscopy (dSTORM, Nanoimager S, Oxford Nanoimaging), with staining conducted according to the manufacturer's protocol using the EV Profiler 2 kit. All three liposomes were stained with membrane dyes in the kit, followed by ultrafiltration purification. EVs were labeled using the Tetraspanin Trio Detection in the kit and were purified by ultrafiltration. Stained liposomes and EVs were mixed at a 1:1 ratio (number of particles), incubated at 37 °C for 2 h in PBS buffer containing polyethylene glycol (PEG8000, 5%, m/V), and analyzed using super‐resolution microscopy (Nanoimager S, Oxford Nanoimaging). Two thousand images were acquired at 50% power on the 561 nm laser (EVs) and 488 nm laser (liposomes) for localization, respectively. Colocalization was analyzed using the CODI platform (https://alto.codi.bio/). Liposomes were labeled with green pseudocolor, while EVs were labeled with orange pseudocolor. For DLS, each of the three liposomes was mixed with extracellular vesicles in a 1:1 ratio (number of particles) and incubated at 37 °C for 2 h. The particle size of the mixture was then measured using a Zetasizer Nano ZS90 (Malvern Panalytical). To further investigate the optimal composition, different concentrations of polyethylene glycol (PEG8000, m/V) and NaCl (mM) were employed. Size changes during the reaction were obtained via DLS (Malvern Panalytical), while fluorescence intensity variations were measured using a microplate reader (Tecan) and a fluorescence spectrometer (Horiba Scientific). To investigate the membrane fusion efficiency between liposomes with different charges and EVs, EVs derived from Raji cells were stained with membrane dyes DiO (10 µM) and DiI (10 µM) and subsequently purified by ultrafiltration. The labeled EVs were mixed with three types of liposomes at a 1:1 ratio (number of particles) and incubated at 37 °C for 2 h in PBS containing polyethylene glycol (PEG8000, 5%, m/v). Fluorescence intensity variations were measured using a microplate reader (Tecan) and a fluorescence spectrometer (Horiba Scientific). Membrane fusion efficiency was determined by measuring changes in DiO fluorescence intensity before and after fusion. The fluorescence intensity of DiO was denoted as F_DiO_. Fusion efficiency was calculated using the following equation:

(2) $$\mathrm{Membrane}\;\mathrm{fusion}\;\mathrm{efficiency}=\left(F_{n\;}-\;F_{0}\right)\;/\;\left(F_{\mathrm{DiO}\;}-\;F_{0}\right)$$

where F_0_ and F_n_ represent the fluorescence intensity before and after fusion, respectively.

### Collection and Preparation of Clinical Samples

Clinical experiments were approved by the Ethics Committee of the Zhongda Hospital of Southeast University (No. 2023ZDSYLL238) and were conducted in accordance with the ethical standards. Blood samples were collected from healthy volunteers and lymphoma patients at the Zhongda Hospital of Southeast University with informed consent. Sample processing was strictly performed according to the steps recommended in previously reported studies.^[^ ^26^ ^]^ Blood was collected from fasting individuals by venipuncture in the early morning. Blood samples were collected in EDTA tubes and processed by centrifugation within 1 h. Samples were centrifuged at 1000 g for 10 min and 2500 g for 10 min at room temperature to extract plasma, and were stored at −80 °C for further use.

### EV miRNA Analysis of Plasma Samples

Extracted plasma samples were utilized for further analysis. For the standardized analysis of plasma EV miRNA expression levels, EVs were first isolated by UC as described previously and characterized by NTA and TEM. Subsequently, EV miRNA extraction and miRNA expression level identification based on reverse transcription and gold standard qPCR were performed (Servicebio Technology). Relative expression level was calculated using the following equation: relative expression level = 2^−ΔΔCt^. For performing multiplexed detection using the developed method, plasma samples were first diluted at different ratios to explore optimal conditions. Under conditions of consistent total volume, fluorescence intensity was measured using a microplate reader (Tecan) after a reaction time of 2 h. Subsequently, ensuring identical sample volumes for patients and HC (20 µL), ALs containing probes for three targets (miR‐155‐5p, miR‐125b‐5p, miR‐21‐5p) were added, respectively. Fluorescence intensity of the solution was acquired using a microplate reader (Tecan).

### Statistical Analysis

Two‐tailed Student's t‐test was used to evaluate statistical mean differences between two groups. The receiver operating characteristic curve (ROC) was used to measure analytical metrics performance. All statistical analyses were performed using the GraphPad software (Prism 10) and OriginLab (Origin 2024b). *P* < 0.05 was regraded statistically significant.

## Conflict of Interest

The authors declare no conflict of interest.

## Supporting information



Supporting Information

## Data Availability

The data that support the findings of this study are available from the corresponding author upon reasonable request.
