# Supplementary material for: Anionic Liposomes as Optimal Membrane Fusion Carriers Enabling in Situ Multiplexed Detection of Extracellular Vesicle MicroRNAs
Source: Adv Sci (Weinh). 2025 Dec 8;13(10):e19758. doi: 10.1002/advs.202519758 (PMC12915101; doi:10.1002/advs.202519758)
Supplement: Supplementary file 1 — Supporting Information [file ADVS-13-e19758-s001.pdf]

Supporting Information

Anionic Liposomes as Optimal Membrane Fusion Carriers Enabling In Situ Multiplexed Detection of Extracellular Vesicle MicroRNAs

Jingyuan Ma,[a] Xiao Wang,[a] Yuhan Cai,[a] Guancheng Wang,[a] Mingze Lu,[a] Kaizheng Feng,[a] Ying Zhao,[a] Xue Wu,[b] Xiaoping Zhang,[b] Haoan Wu,[a] Wei Yu,[c] Ming Ma,*[a] Zheng Ge,*[b] Yu Zhang*[a]

*[a] J. Ma, X. Wang, Y. Cai, G. Wang, M. Lu, K. Feng, Y. Zhao, H. Wu, M. Ma, Y. Zhang
State Key Laboratory of Digital Medical Engineering, Jiangsu Key Laboratory for Biomaterials and Devices, School of Biological Science and Medical Engineering
Southeast University
Nanjing, Jiangsu 210096, P. R. China
E-mail: zhangyu@seu.edu.cn; maming@seu.edu.cn

*[b] X. Wu, X. Zhang, Z. Ge
Department of Hematology, Zhongda Hospital, School of Medicine, Institute of Hematology
Zhongda Hospital Southeast University
Nanjing, Jiangsu 210009, P. R. China
E-mail: gezheng2008@163.com

[c] W. Yu
Department of Medical Laboratory, Taikang Xianlin Drum Tower Hospital
Nanjing University School of Medicine
Nanjing, Jiangsu 210000, P. R. China

Table of contents

1. **Experimental Instruments and Procedures**

2. **Supplementary Figures:**

   Figure S1 to Figure S19

3. **Supplementary Table:**

   Tables S1 to Table S4

Experimental Instruments and Procedures

Chemicals

All the chemicals were of analytical grade and were used without further purification. DNA marker, 6 × TriTrack DNA loading buffer (GeneRuler DNA Ladder), and fetal bovine serum (FBS) were provided by Thermo Fisher Scientific Co., Ltd. (Shanghai, China). DMPC (1,2-Dimyristoyl-sn-glycero-3-phosphocholine), DOTMA (1,2-di-O-octadecenyl-3-trimethylammonium propane), and cholesterol were provided by AVT (Shanghai) Pharmaceutical Tech Co., Ltd. DPPG (1,2-Dipalmitoyl-sn-glycero-3-phospho-rac-[1-glycerol] sodium) was obtained from Xi'an Ruixi Biotechnology Co., Ltd. RPMI-1640 was purchased from Jiangsu Keygen Biotech Corp., Ltd. DiO, DiI was obtained from Beyotime Biotechnology. Agarose was purchased from Biowest Agarose. Phosphate buffer solution (PBS, 0.01 M) was purchased from Gibco of Thermo Fisher Scientific Co., Ltd. EV Profiler 2 for direct stochastic optical reconstruction microscopy (dSTORM) was obtained from Oxford Nanoimaging. All DNA and RNA used in the work were synthesized and purified by Sangon Biotech (Shanghai) Co., Ltd., and their sequences are listed in Table S1. Fluorescence quantitative PCR (qPCR) and western blot were performed by Wuhan Servicebio Technology Co., Ltd.

Instruments

Microfluidic chip (fish bone microhybrid liposome chip, ZX-LS-31) was purchased from Suzhou Cchip Scientific Instrument CO., Ltd.

Nanoparticle tracking analysis (ZetaView, Particle Metrix).

Particle size and zeta potential analyzer (Zetasizer Nano ZS90, Malvern Panalytical).

Fourier transform infrared spectroscopy (FT-IR, Nicolet iS10, Thermo Fisher Scientific).

Transmission electron microscopy (TEM, Talos F200X, Thermo Fisher Scientific).

Fluorescence spectrometer (Fluoromax-4, Horiba Scientific).

Microplate readers (Infinite M200, Tecan).

Gel Imaging System (ImageQuant LAS 500, GE Healthcare).

Ultracentrifuge (Optima MAX-XP, Beckman Coulter).

Centrifuge (5810R, Eppendorf).

Direct stochastic optical reconstruction microscopy (dSTORM, Nanoimager S, Oxford Nanoimaging)

Calculation process of the detection limit

The LOD (limit of detection) is calculated based on the LOD definition of the International Union of Pure and Applied Chemistry (IUPAC). LOD refers to the lowest concentration (CL) corresponding to the minimum analytical signal (XL) that can be detected. When calculating the LOD of the sensor, first perform ten parallel measurements on a blank sample to obtain the corresponding average value (Xb) and standard deviation (σ), and the minimum value is calculated using formula S1.

$$XL = Xb + k \times \sigma \tag{S1}$$

In this formula, Xb represents the average value of the blank sample; σ represents the standard deviation of the blank sample; k is a numerical factor selected based on the desired confidence level. IUPAC recommends using k = 3 as the standard for calculating the detection limit, which corresponds to approximately a 90% confidence level.

Artificial intelligence-assisted indicator weight calculation

The indicator calculation is implemented through the following code. The code is executed and analyzed through PyCharm (2024.3.1.1, Professional Edition, JetBrains).

```
import numpy as np
import pandas as pd
from sklearn.model_selection import train_test_split
from sklearn.neural_network import MLPClassifier
group1_patients = [data1,data2,…,]
group1_healthy = []
group2_patients = []
group2_healthy = []
group3_patients = []
group3_healthy = []
X = np.concatenate([
    np.column_stack([group1_patients, group2_patients, group3_patients]),
    np.column_stack([group1_healthy, group2_healthy, group3_healthy])
], axis=0)
y = np.concatenate([np.ones(len(group1_patients)), np.zeros(len(group1_healthy))])
X_train, X_test, y_train, y_test = train_test_split(X, y, test_size=0.2, random_state=42)
mlp = MLPClassifier(hidden_layer_sizes=(10,), max_iter=500, random_state=42)
mlp.fit(X_train, y_train)
weights = np.mean(np.abs(mlp.coefs_[0]), axis=1)
weights /= weights.sum()
for i, weight in enumerate(weights):
    print(f"Group {i + 1} weight: {weight:.4f}")
adjusted_patients = np.dot(X[y == 1], weights)
adjusted_healthy = np.dot(X[y == 0], weights)
adjusted_data = pd.DataFrame({
    "Adjusted Patients": adjusted_patients,
    "Adjusted Healthy": adjusted_healthy
})
output_file = ""
adjusted_data.to_excel(output_file, index=False)
print(f"saved {output_file}")
```

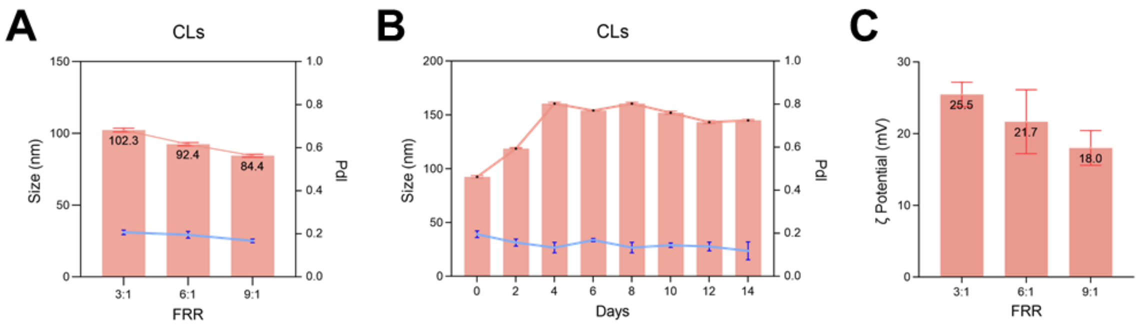

Figure S1. Synthesis and stability characterization of cationic liposomes (CLs). (A) Hydrodynamic size of CLs with different flow ratios. **(B)** Stability of CLs over a period of 14 days (FRR = 6:1). **(C)** ζ potential of CLs with different flow ratios.

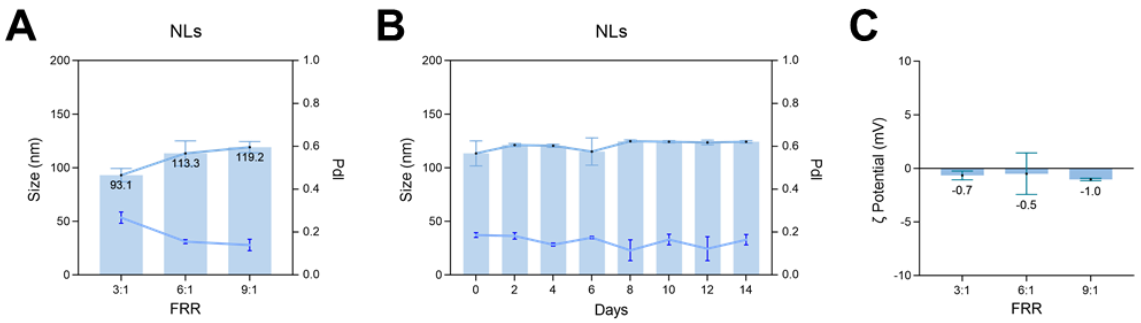

Figure S2. Synthesis and stability characterization of neutral liposome (NLs). (A) Hydrodynamic size of NLs with different flow ratios. **(B)** Stability of NLs over a period of 14 days (FRR = 6:1). **(C)** ζ potential of NLs with different flow ratios.

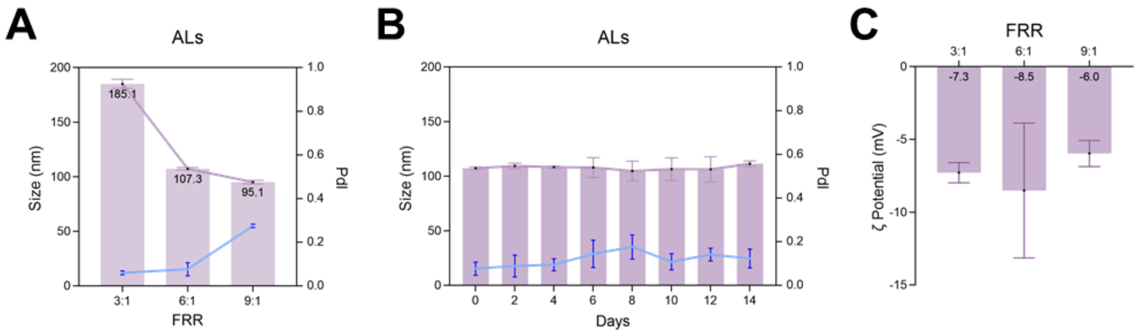

Figure S3. Synthesis and stability characterization of anionic liposome (ALs). (A) Hydrodynamic size of ALs with different flow ratios. **(B)** Stability of ALs over a period of 14 days (FRR = 6:1). **(C)** ζ potential of ALs with different flow ratios.

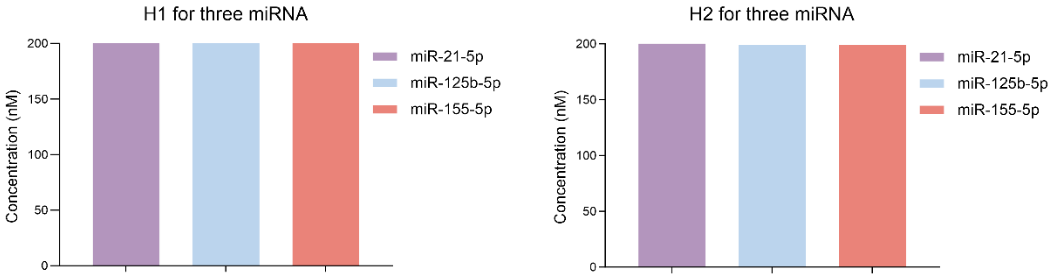

Figure S4. Prediction of the concentration of 200 nM free nucleic acid forming hairpin probes by Nupack.

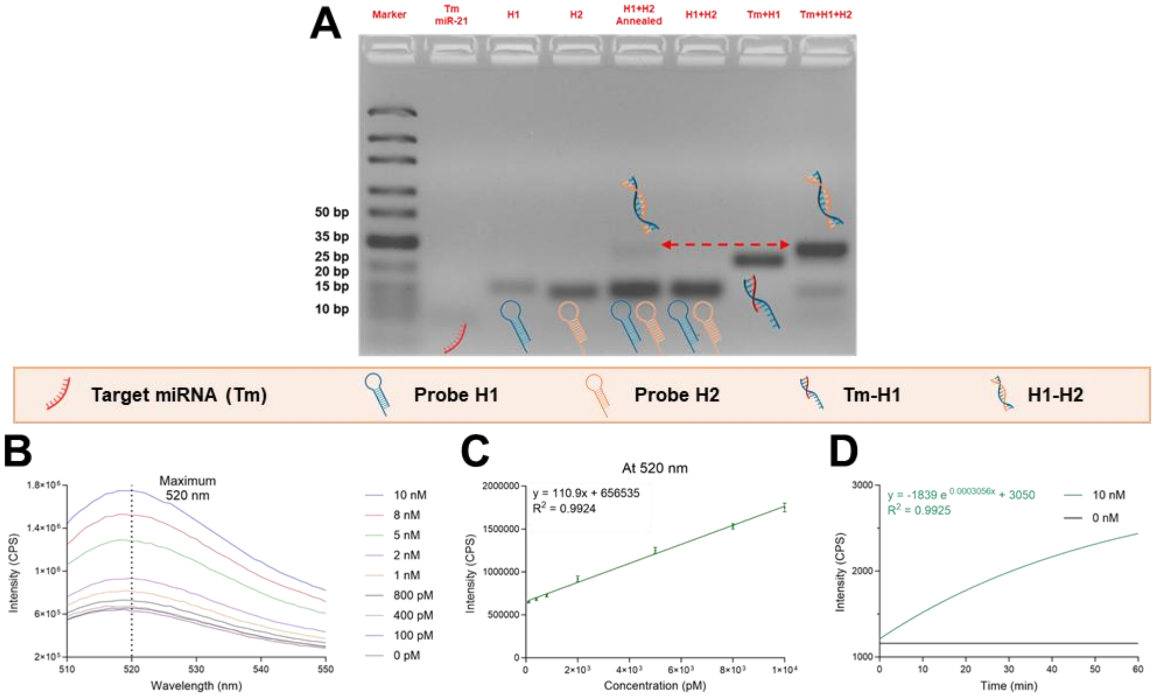

Figure S5. Toehold-mediated strand displacement reaction for the detection of the target miRNA (miR-21-5p, Tm). (A) Agarose gel electrophoresis analysis with different nucleic acid molecular inputs (Tm, H1, H2, annealed H1+H2, H1+H2, Tm+H1, and Tm+H1+H2 are shown in lanes 2–8, respectively, and the marker is shown in lane 1). **(B, C)** Fluorescence spectra of the reaction system corresponding to different concentrations of Tm input **(B)** and linear relationship at the maximum emission wavelength **(C)**. **(D)** Fluorescence measurement of strand displacement reactions triggered by miR-21-5p.

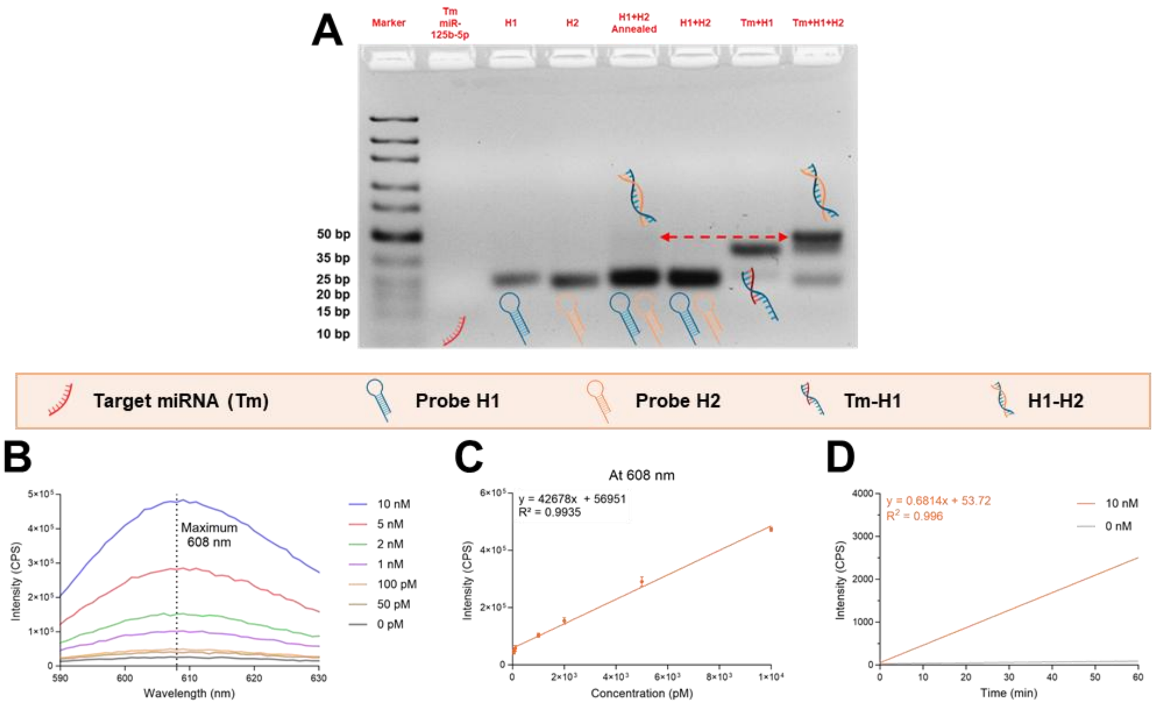

Figure S6. Toehold-mediated strand displacement reaction for the detection of the target miRNA (miR-125b-5p, Tm). (A) Agarose gel electrophoresis analysis with different nucleic acid molecular inputs (Tm, H1, H2, annealed H1+H2, H1+H2, Tm+H1, and Tm+H1+H2 are shown in lanes 2–8, respectively, and the marker is shown in lane 1). **(B, C)** Fluorescence spectra of the reaction system corresponding to different concentrations of Tm input **(B)** and linear relationship at the maximum emission wavelength **(C)**. **(D)** Fluorescence measurement of strand displacement reactions triggered by miR-125b-5p.

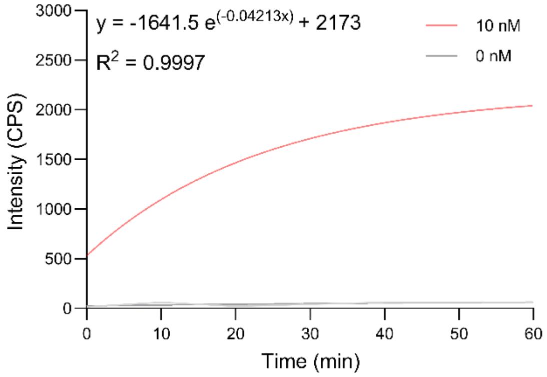

Figure S7. Fluorescence measurement of strand displacement reactions triggered by miR-155-5p.

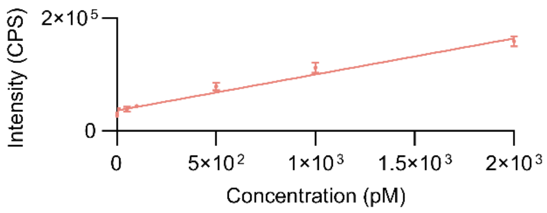

Figure S8. **The corresponding linear correlation between fluorescence intensity (at the maximum emission wavelength) and Tm (miR-155-5p) concentration.**

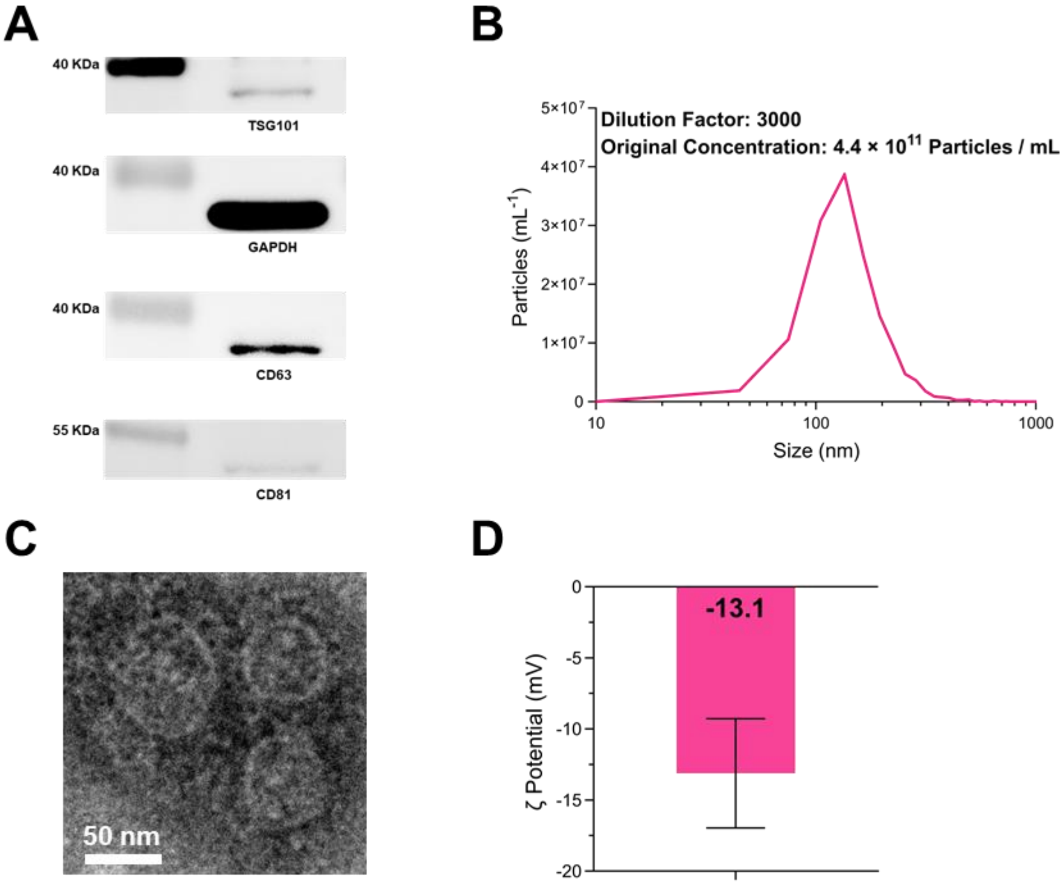

Figure S9. Characterization of extracellular vesicles (EVs) derived from Raji. (A) Western blotting analysis of TSG101, GAPDH, CD63, and CD81 expression levels. **(B)** Nanoparticle tracking analysis of EVs. **(C)** Transmission electron microscope image of EVs. **(D)** ζ potential characterization of EVs.

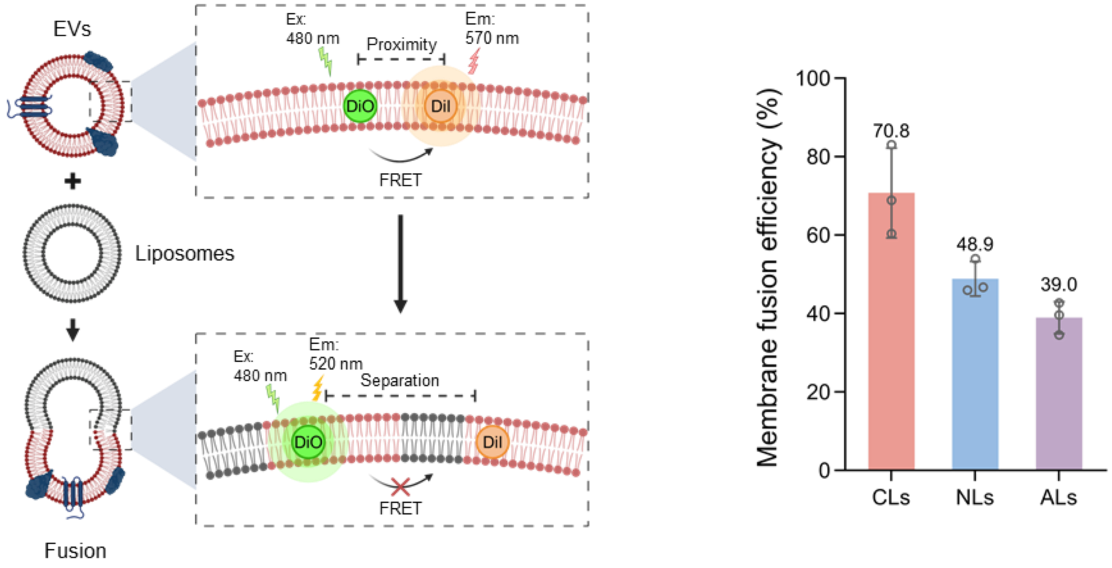

Figure S10. Determination of fusion efficiency for three liposomes based on fluorescence resonance energy transfer.

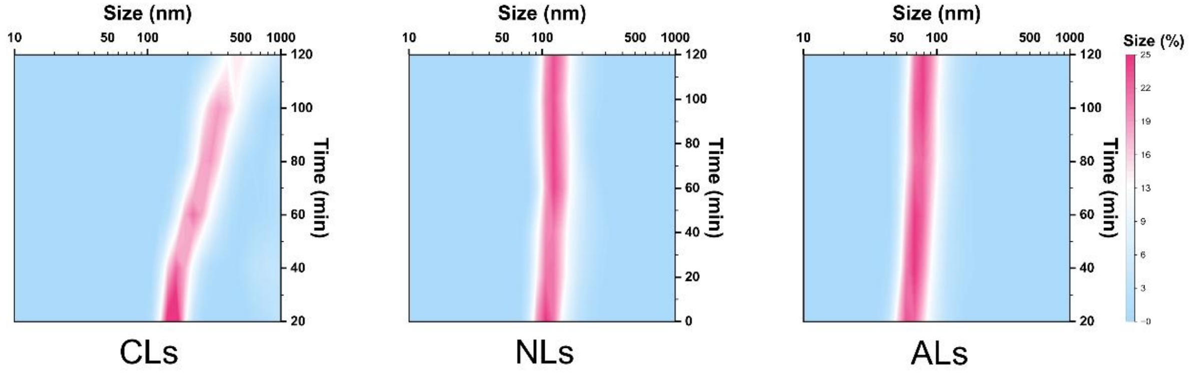

Figure S11. ***In situ*** **hydrodynamic size changes of Raji cell-derived EVs with probe-loaded cationic liposomes (CLs), neutral liposomes (NLs), and anionic liposomes (ALs) in the presence of 75 nM additional salt ions and in the absence of polyethylene glycol (PEG8000).**

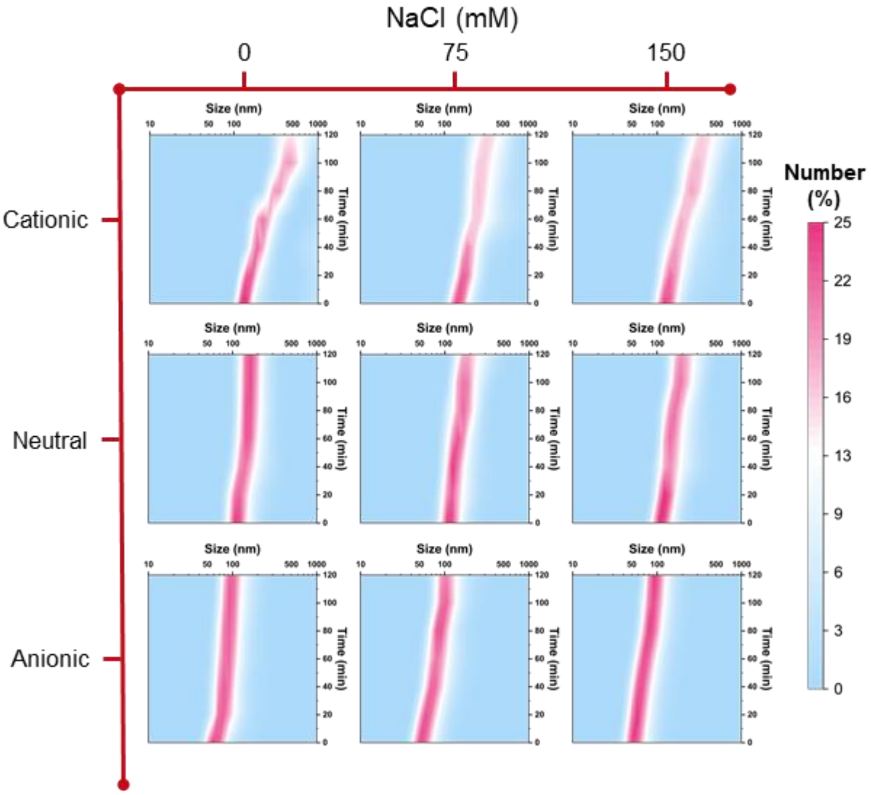

Figure S12. Optimization of membrane fusion conditions and liposome screening. *In situ* hydrodynamic size profiles of Raji cell-derived extracellular vesicles (EVs) with cationic liposomes (CLs), neutral liposomes (NLs), and anionic liposomes (ALs) at different salt ion concentrations.

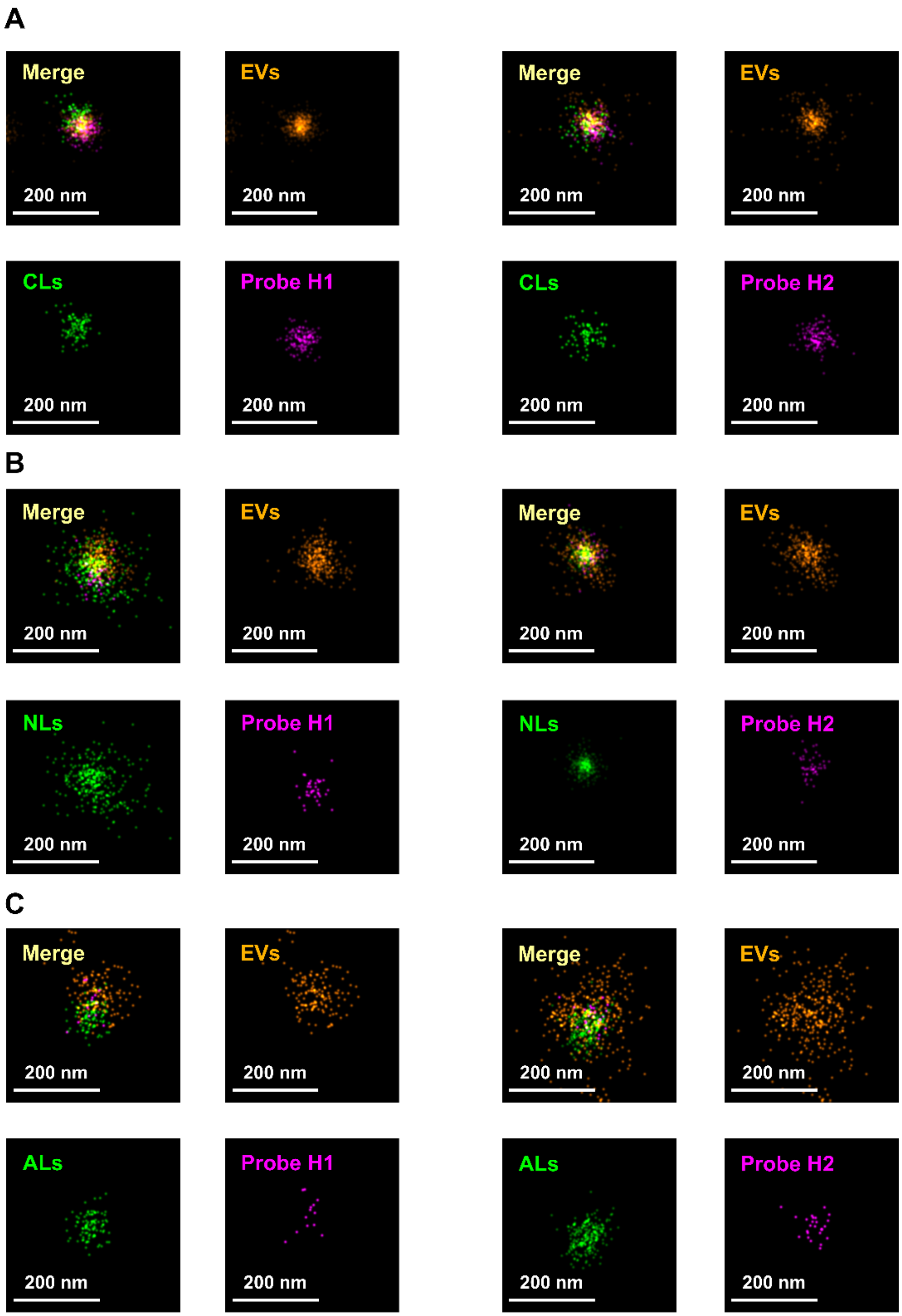

Figure S13. Characterization of fusion between three composite liposomes encapsulated with probes H1 or H2 and EVs.

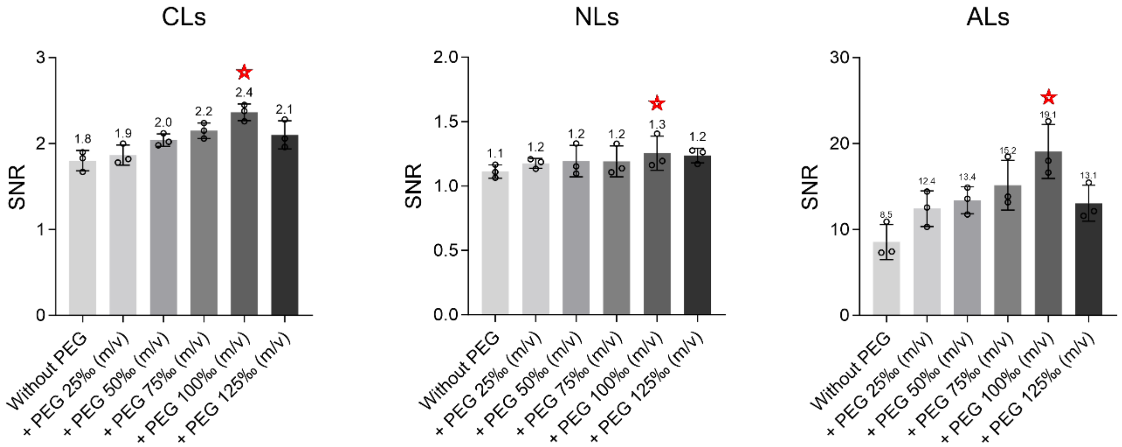

Figure S14. Signal-to-noise ratio (SNR) of cationic liposomes (CLs), neutral liposomes (NLs), and anionic liposomes (ALs) with different PEG concentrations. All data were collected at the end of the reactions. All data are presented as mean ± standard deviation (SD) from three independent experiments.

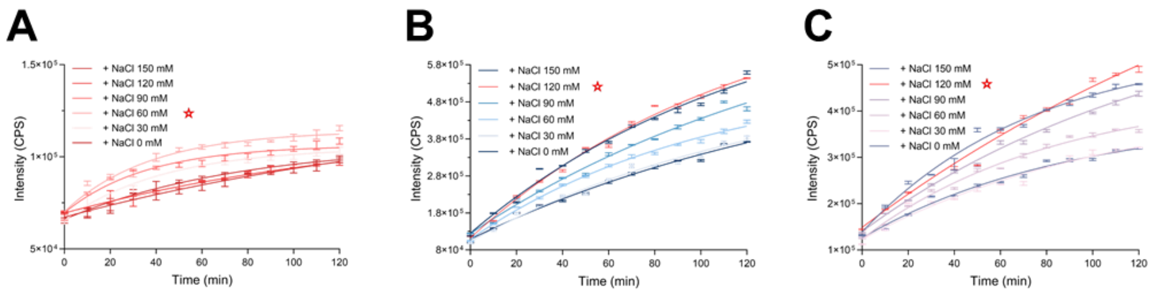

Figure S15. Kinetic profiles of EVs with cationic liposomes (A), neutral liposomes (B), and anionic liposomes (C) at different salt ion concentrations. All data are presented as mean ± standard deviation (SD) from three independent experiments.

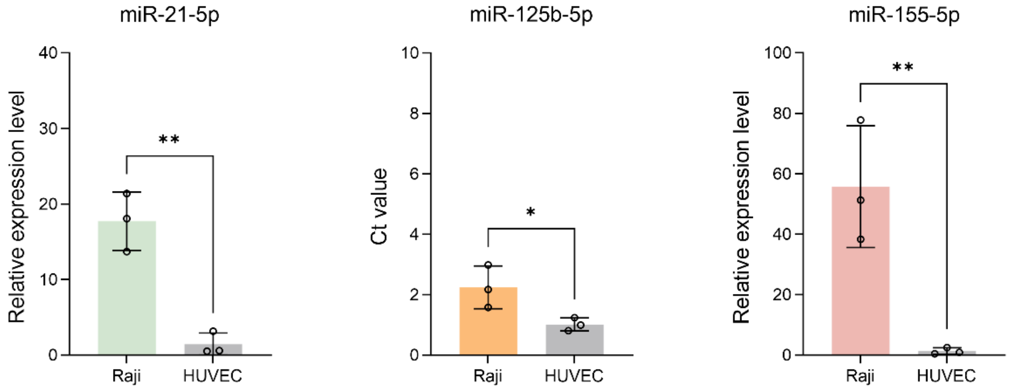

Figure S16. Fluorescence quantitative PCR characterization of miRNA expression levels within Raji cells and HUVEC-derived EVs. All data are presented as mean ± SD from three independent experiments. * $p < 0.05$, ** $p < 0.01$.

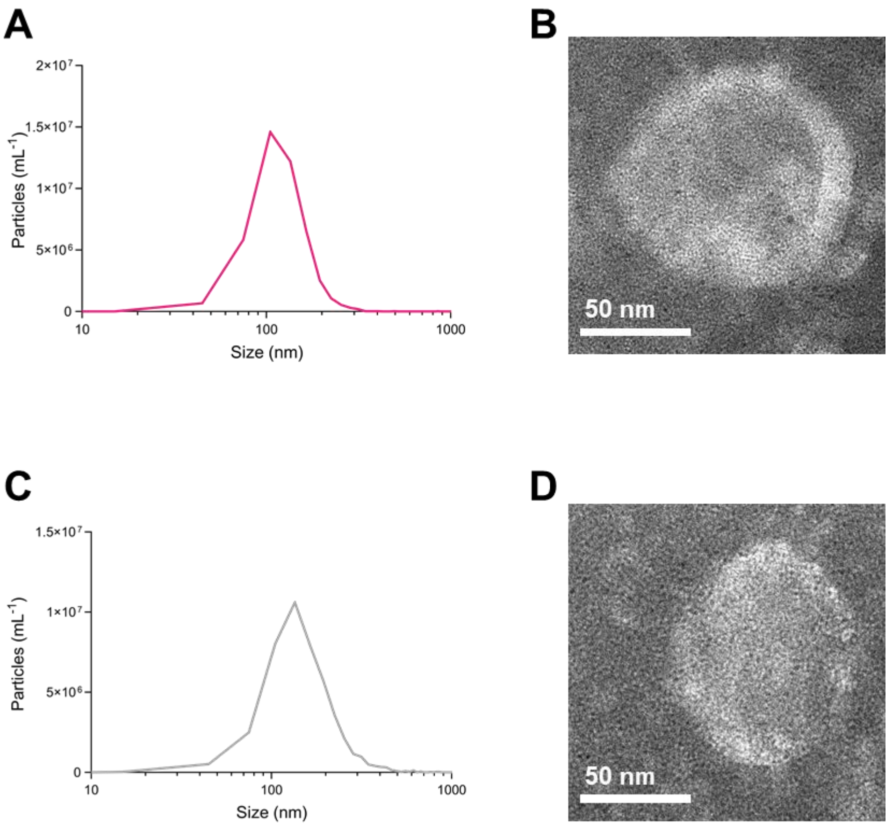

Figure S17. Characterization of EVs derived from plasma samples purified by ultracentrifugation. Nanoparticle tracking analysis (NTA) **(A)** and transmission electron microscopy (TEM) **(B)** characterization of EVs from patient plasma, and NTA **(C)** and TEM **(D)** characterization of EVs from HC plasma.

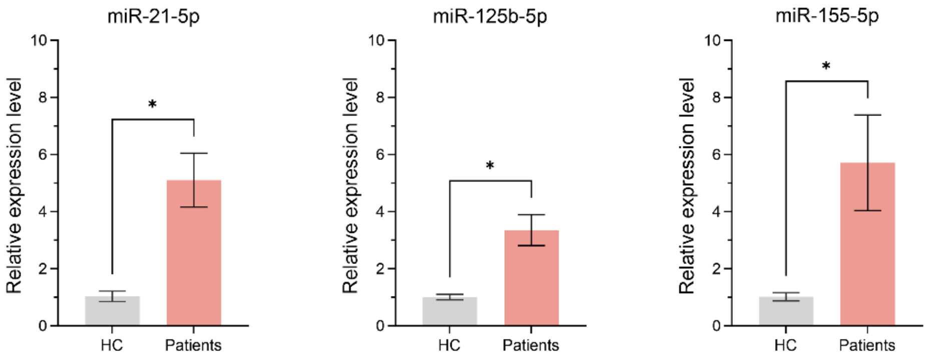

Figure S18. Fluorescence quantitative PCR characterization of plasma-derived extracellular vesicles of lymphoma patients and healthy controls (HC). All data are presented as mean ± standard deviation (SD) from three independent experiments. * $p < 0.05$.

Reference	Detection Principle	Carrier	RNA Extraction	Enzyme Dependence	Complexity	Scalability
[25]	Magnetic nanoparticle, CRISPR/Cas12a, DSN, and electrochemical based	Magnetic nanoparticle	Required	Yes	High	Low
[36]	Cell membrane vesicle, TMSD, and electro-chemical based	Cell membrane vesicle	Required EV lysis for electrochemical	No	Moderate	Low
[41]	Cationic core-shell-corona structure, CHA, and CRISPR-Cas12a based	Cationic core-shell-corona structure	Not required	Yes	High	Moderate
[43]	MOF, cationic liposome, MB, and DNAzyme based	MOF and cationic liposome	Not required	No	High	Low
[44]	Membrane fusion and CRISPR/Cas13a based	Cationic liposome	Not required	Yes	Moderate	Moderate
[45]	Microfluidic droplet reactor, cationic liposome, and MB based	Cationic liposome	Not required	No	Moderate	High
**Our method**	**Membrane fusion and TMSD based**	**Anionic liposome**	**Not required**	**No**	**Low**	**High**

Figure S19. Comparison of EValarm with other detection systems. DSN: duplex-specific nuclease; TMSD: toehold-mediated strand displacement; CHA: catalytic hairpin assembly; MOF: metal-organic framework; MB: molecular beacon.

Table S1. Sequences of oligonucleotides used in this work.

Oligonucleotides	Sequence (from 5' to 3')
miR-155-5p	UUAAUGCUAAUCGUGAUAGGGGU
H1	ACCCCTATCACGATTAGCATTAACCATGTGTAGATTAATGCTAA
H2	GCATTAATCTACACATGGTTAATGCTAATCGTGCATGTGTAGA
H1 Cy5	Cy5-ACCCCTATCACGATTAGCATTAACCATGTGTAGATTAATGCTAA
H2 Cy5	Cy5-GCATTAATCTACACATGGTTAATGCTAATCGTGCATGTGTAGA
H2 dual-labeled	Cy5-GCATTAATCTACACATGGTTAATGCTAATCGTGCATGTGTAGA-BHQ
miR-125b-5p	UCCCUGAGACCCUAACUUGUGA
H1	TCACAAGTTAGGGTCTCAGGGACCATGTGTAGATCCCTGAGAC
H2	TCAGGGATCTACACATGGTCCCTGAGACCCTAACCATGTGTAGA
H1 ROX	ROX-TCACAAGTTAGGGTCTCAGGGACCATGTGTAGATCCCTGAGAC
H2 ROX	ROX-TCAGGGATCTACACATGGTCCCTGAGACCCTAACCATGTGTAGA
H2 dual-labeled	ROX-TCAGGGATCTACACATGGTCCCTGAGACCCTAACCATGTGTAGA-BHQ

miR-21-5p	UAGCUUAUCAGACUGAUGUUGA
H1	TCAACATCAGTCTGATAAGCTACCATGTGTAGATAGCTTATCAG
H2	GATAAGCTATCTACACATGGTAGCTTATCAGACTCCATGTGTAGA
H1 FAM	FAM-TCAACATCAGTCTGATAAGCTACCATGTGTAGATAGCTTATCAG
H2 FAM	FAM-GATAAGCTATCTACACATGGTAGCTTATCAGACTCCATGTGTAGA
H2 dual-labeled	FAM-GATAAGCTATCTACACATGGTAGCTTATCAGACTCCATGTGTAGA-BHQ
Mismatch Tm for miR-155-5p	
Mismatch 1	UCCCUGAGACCCUAACUUGUGA
Mismatch 2	UAGCUUAUCAGACUGAUGUUGA

Table S2. Limit of detection for Raji cell-derived EVs corresponding to liposomes with different charge.

**Types**	**Limit of detection (EVs / mL)**
CLs	4×10^{10}
NLs	1.7×10^{9}
**ALs**	$\mathbf{6.1 \times 10^{7}}$

Table S3. Weights of indicators after training.

**Indicator**	**Weight**
miR-155-5p	0.3655
miR-125b-5p	0.3612
miR-21-5p	0.2733

Table S4. Comparison of the detection efficacy of a single indicator compared to a weight-adjusted three-indicator approach.
(Gray squares represent test results inconsistent with clinical results, and green squares represent consistent results.)

Clinical sample	HC 1	HC 2	HC 3	HC 4	HC 5
Single indicator					
Weighted-adjusted three indicators					
Clinical sample	Lymphoma patient 1	Lymphoma patient 2	Lymphoma patient 3	Lymphoma patient 4	Lymphoma patient 5
Single indicator					
Weighted-adjusted three indicators					
